# The date/delay effect in intertemporal choice: A combined fMRI and eye‐tracking study

**DOI:** 10.1002/hbm.26585

**Published:** 2024-02-24

**Authors:** Kristof Keidel, Rebekka Schröder, Peter Trautner, Alexander Radbruch, Carsten Murawski, Ulrich Ettinger

**Affiliations:** ^1^ Department of Psychology University of Bonn Bonn Germany; ^2^ Department of Finance, Centre for Brain, Mind and Markets The University of Melbourne Carlton Victoria Australia; ^3^ Core Facility Human 3T MRI University of Bonn Bonn Germany; ^4^ Clinic of Neuroradiology University Hospital Bonn Germany; ^5^ Clinical Neuroimaging, German Center for Neurodegenerative Diseases (DZNE) Bonn Germany

**Keywords:** date/delay effect, episodic thinking, eye‐tracking, fMRI, precuneus, temporal discounting

## Abstract

Temporal discounting, the tendency to devalue future rewards as a function of delay until receipt, is influenced by time framing. Specifically, discount rates are shallower when the time at which the reward is received is presented as a date (date condition; e.g., June 8, 2023) rather than in delay units (delay condition; e.g., 30 days), which is commonly referred to as the date/delay effect. However, the cognitive and neural mechanisms of this effect are not well understood. Here, we examined the date/delay effect by analysing combined fMRI and eye‐tracking data of *N* = 31 participants completing a temporal discounting task in both a delay and a date condition. The results confirmed the date/delay effect and revealed that the date condition led to higher fixation durations on time attributes and to higher activity in precuneus/PCC and angular gyrus, that is, areas previously associated with episodic thinking. Additionally, participants made more comparative eye movements in the date compared to the delay condition. A lower date/delay effect was associated with higher prefrontal activity in the date > delay contrast, suggesting that higher control or arithmetic operations may reduce the date/delay effect. Our findings are in line with hypotheses positing that the date condition is associated with differential time estimation and the use of more comparative as opposed to integrative choice strategies. Specifically, higher activity in memory‐related brain areas suggests that the date condition leads to higher perceived proximity of delayed rewards, while higher frontal activity (middle/superior frontal gyrus, posterior medial frontal cortex, cingulate) in participants with a lower date/delay effect suggests that the effect is particularly pronounced in participants avoiding complex arithmetic operations in the date condition.

## Graphical abstract

We used eye‐tracking and fMRI to examine mechanisms of time framing in intertemporal choice. Results revealed that date framing compared to delay unit framing led to higher fixation durations on time attributes and higher brain activity in areas associated with episodic thinking, consistent with hypotheses positing differential time estimation.

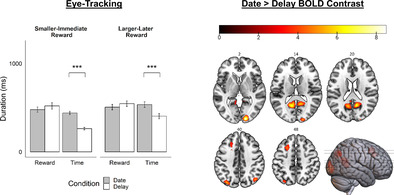

**Practitioner Points**

- In intertemporal choices, temporal discounting is reduced when the timing of rewards is presented in terms of dates rather than delay units (date/delay effect).
- The date condition is associated with longer fixation durations on time compared to reward attributes, more comparative eye movements between time attributes and higher neural activity in areas associated with episodic future thinking (precuneus, posterior cingulate cortex, angular gyrus), in line with hypotheses positing differences in time estimation.
- People with a lower date/delay effect had relatively higher frontal activity (e.g., middle/superior frontal gyrus) in the date condition, suggesting that doing mental math in that condition may undermine its effectiveness.

## 1 INTRODUCTION

Choosing between smaller rewards available sooner and larger rewards available later is known as intertemporal choice. When making such choices, people typically prefer smaller but earlier rewards to larger but later rewards, devaluing future rewards as a function of time. This phenomenon is referred to as temporal discounting (Frederick et al., 2002). Higher temporal discounting has been associated with negative life outcomes (e.g., higher credit debts, more risky behaviour) in the general population (Keidel et al., 2021) and has been documented for a broad range of mental disorders (Amlung et al., 2019). Various experimental manipulations have been shown to attenuate temporal discounting (Rung & Madden, 2018; H. Scholten et al., 2019), including the date/delay effect (LeBoeuf, 2006; Read et al., 2005): People discount future rewards less when time until receipt of rewards is framed in terms of dates (date condition; e.g., 8th June 2023) instead of delay units (delay condition; e.g., 30 days). While this effect has been replicated several times (Dshemuchadse et al., 2013; Jiang & Dai, 2021; Keidel, Murawski, & Ettinger, 2023; Keidel, Murawski, Pantelis, & Ettinger, 2023; Naudé et al., 2018), its cognitive and neural underpinnings are still ambiguous. We therefore combined, for the first time, functional magnetic resonance imaging (fMRI) with eye‐tracking to investigate the neural and cognitive mechanisms of the date/delay effect.

### 1.1 Neural mechanisms of intertemporal choice

Neural activity patterns during intertemporal choices have been studied extensively (Frost & McNaughton, 2017; Kable & Glimcher, 2007; Peters & Büchel, 2011). Frost and McNaughton (2017) deduced a cognitive model of intertemporal decision‐making comprising five brain systems, viz. (i) an object representation system responsible for transmitting sensory information (occipital lobe), (ii) a memory‐driven system to derive gain‐related information (memory‐related regions, e.g., medial prefrontal cortex [mPFC], precuneus, medial temporal lobe, lateral temporal cortex and lateral parietal cortex), (iii) a subjective value (SV) computation system (e.g., mPFC, ventral striatum [vS]; also see Bartra et al., 2013), (iv) a comparator system to compare choice alternatives (regions involved in evaluating choice difficulty, e.g., striatal and prefrontal areas, amygdala, and their connectivity) and (v) a motor system to produce and suppress responses (motor areas). In our study, we did not target one specific part of the process (e.g., valuation) but differences between date and delay conditions in intertemporal choice processes as a whole. We were particularly interested in whether activity patterns in the date and delay conditions might differ in the memory‐driven, SV computation or comparator systems.

### 1.2 Potential mechanisms of the date/delay effect

Different cognitive mechanisms of the date/delay effect have been proposed (Dshemuchadse et al., 2013; LeBoeuf, 2006; Read et al., 2005), including the differential time estimation hypothesis (Read et al., 2005; Zauberman et al., 2009), the attention‐focusing or reward‐weighting hypothesis (Keidel, Murawski, & Ettinger, 2023; LeBoeuf, 2006; Read et al., 2005) and the choice strategy hypothesis (Read et al., 2005).

The differential time estimation hypothesis states that time intervals presented in terms of dates are subjectively estimated to be shorter than intervals presented as delay units (Read et al., 2005; Zauberman et al., 2009). Thus, underestimating the waiting time (Ebert & Prelec, 2007; Jiang & Dai, 2021; Zauberman et al., 2009) for the delayed reward in the date compared to the delay condition may lead to less temporal discounting. One reason for this phenomenon could be that time estimation is influenced by the abstractness of time information (Lempert & Phelps, 2016). According to Construal Level Theory (Trope & Liberman, 2003), abstract, high‐level construals have a high psychological distance, while more concrete, lower‐level construals have a low psychological distance. As dates might be more concrete because of their specificity and have potentially higher personal relevance (e.g., triggering more episodic processing) than delay units, the psychological distance might be lower in the date frame, and thus, a date might appear closer to the current day than a specific delay unit. In the context of the systems proposed by Frost and McNaughton (2017), time estimation could be influenced by episodic memory and thinking about the future (Cooper et al., 2013; Peters & Büchel, 2010). Accordingly, differences between conditions should cause divergent activity in the memory‐related or SV systems in response to time information.

Second, the attention‐focusing (Read et al., 2005) or reward‐weighting hypothesis (Keidel, Murawski, & Ettinger, 2023; LeBoeuf, 2006) postulates that when time is given in delay units, attention is drawn to the waiting time, whereas dates draw attention to the time at which a reward is received and to the reward at that time. As such, dates are assumed to lead to a relatively higher weighting of reward than time attributes. This could also be associated with overt shifts of attention toward rewards (Read et al., 2005). However, two recent studies contradict this notion. They found that the date condition led to higher fixation durations on time rather than reward attributes, while there was no change in the number of reward attribute comparisons (Keidel, Murawski, & Ettinger, 2023; Sharma & Khan, 2022). Nevertheless, one of the studies found that reward differences were more important for the ultimate decision, suggesting potential differences in internal weighting processes (Keidel, Murawski, & Ettinger, 2023). At the neural level, a stronger focus on rewards should be associated with divergent signalling in valuation areas such as mPFC or vS in response to reward information. In case of overt attentional shifts, this should also be accompanied by higher fixation durations on reward vs. time attributes and potentially by more saccades between reward attributes (see next paragraph).

Third, in combination with the reward‐focusing hypothesis, the choice strategy hypothesis is based on the assumption that dates impede the calculation of interest rates (Read et al., 2005). This hypothesis is related to the distinction between traditional alternative‐based (Frederick et al., 2002) and more recently introduced attribute‐based models (Amasino et al., 2019; Dai & Busemeyer, 2014; Marzilli Ericson et al., 2015; Read et al., 2013; Scholten & Read, 2010) of intertemporal choice. Specifically, in traditional intertemporal choice tasks with delay units, reward values and delay units are integers that can be combined to estimate SVs of choice options (integrative strategy), which can then be compared in a common neural currency (Levy & Glimcher, 2012; see Hayden & Niv, 2021, for a recent challenge). This procedure follows alternative‐based models, given that SVs of options are computed and compared to derive a decision (Frederick et al., 2002). Interestingly, with regard to the date condition, estimating SVs of choice options is not directly possible. Instead, people need to either transform dates to delay units to be able to continue using the integrative strategy or they need to adapt their strategy. It has been suggested that they might change from integrating reward and time information to comparing attributes separately (comparative strategy) (Amasino et al., 2019; Fisher, 2021; Reeck et al., 2017) and deciding based on the attribute type that is considered more important, which is thought to be the reward (Read et al., 2005). This reflects an implementation of the attribute‐based model, that is, differences in attributes are computed and the decision is based on the more favourable difference (Amasino et al., 2019; Dai & Busemeyer, 2014; Marzilli Ericson et al., 2015; Read et al., 2013; M. Scholten & Read, 2010). Indeed, a higher number of saccades between time attributes have been found in the date condition (Keidel, Murawski, & Ettinger, 2023). Such a change towards a comparative strategy could speak for a change in attentional search and a stronger reward focus (see above). It should be observable in eye‐tracking behaviour (i.e., an increase in the number of saccades comparing attributes of the same type between options relative to the number of saccades between reward and time of one option). Importantly, the idea of different strategy uses in combination with the potential reward focus may reflect more generally that the date condition prompts participants to calculate less in the date compared to the delay condition and take a more intuitive approach to intertemporal decision‐making (e.g., focusing more on episodic processes). A less arithmetic approach may then cause differences between conditions in the activity in prefrontal parts of the comparator system.

Finally, factors that can explain interindividual differences in the magnitude of the date/delay effect are largely unclear. Previous studies suggest that in impulsive individuals, for example, people with substance use disorder (Klapproth, 2012) and those showing steeper discounting rates in the traditional delay condition (Keidel, Murawski, Pantelis, & Ettinger, 2023), discounting is reduced more by the date manipulation (compared to the delay condition). Therefore, differences in impulsivity traits, commonly assessed by questionnaires such as the UPPS‐P Impulsive Behaviour Scale (Lynam et al., 2006; Whiteside & Lynam, 2001), would be expected to be associated with the date/delay effect.

Here, we used fMRI to compare neural activity between date and delay conditions to draw conclusions about the cognitive and neural mechanisms of the date/delay effect and the mechanisms involved in time processing in intertemporal decision‐making. We hypothesised that temporal discounting would be lower in the date compared to the delay condition, thereby replicating the date/delay effect. Furthermore, since our study was the first to use fMRI to investigate the date/delay effect and, as discussed above, a wide range of cognitive and neural mechanisms may potentially be involved, we planned to jointly examine the neural and oculomotor mechanisms of the date/delay effect applying an explorative analysis approach. Generally, for the eye‐tracking data, we expected to find the same effects as in a previous experiment (Keidel, Murawski, & Ettinger, 2023). Finally, we explored whether the date/delay effect or its neural correlates would be associated with questionnaire‐assessed interindividual differences in levels of impulsivity.

## 2 MATERIALS AND METHODS

The study was preregistered at https://osf.io/d9u74. Study materials, behavioural data, analysis scripts and supplementary material are accessible at https://osf.io/td5gf/.

### 2.1 Sample

We based our intended final sample size on an a priori power analysis in G*Power 3.1 (Faul et al., 2009). Specifically, we determined the sample size needed to reveal the date/delay effect (paired *t*‐test) with 80% power, expecting an effect size of *d* = 0.45 (see Hedge's *g* in Rung & Madden, 2018) and assuming an alpha‐level of .05 (one‐tailed). This analysis yielded a target sample size of *N* = 32.

Participants were drawn from the student population. They had to be 18–35 years old, male or female, right‐handed, healthy and of good command of German language. Additionally, for valid eye‐tracking measurements, they needed to have normal or corrected‐to‐normal eyesight (contact lenses). Exclusion criteria comprised a current diagnosis of a psychiatric disorder, a current diagnosis or history of neurological disorders, a current diagnosis or history of psychotic disorders, learning disabilities, history of loss of consciousness for more than 5 min, history of alcohol or drug abuse within the last 12 months, serious physical illness or consummation of any prescription or over‐the‐counter medication 3 days previous to the examination (apart from contraceptives, thyroid or vitamin medications). Participants were also excluded if they had any condition making them unsuitable for fMRI (i.e., claustrophobia, metalliferous implant, large tattoos on the upper half of the body, history of welding work, injury or disease of the inner ear with loss of hearing, pregnancy, currently breastfeeding a baby, history of any heart or head surgery). Additionally, we administered an intertemporal choice task in a laboratory screening before the fMRI session to estimate individual discount rates (see Section 2.5.1), ensuring that the 95% credible interval of the discount rate (per annum) was not entirely below 0, did not include 0 or was not entirely above 2. Meeting any of these criteria would have led to the participant's exclusion, which never occurred. Inclusion and exclusion criteria were assessed in an online pre‐screening and a laboratory screening (see Section 2.2).

As planned, the sample consisted of *N* = 32 participants (16 male, 16 female). One male participant had to be excluded because of excessive head movement, so the final sample comprised *N* = 31 participants (15 male, 16 female) aged 19–29 years (*M* = 23.26, SD = 3.19). All participants were students and had completed at least upper secondary education.

### 2.2 Study procedures

All study procedures were approved by the ethics committee of the Department of Psychology at the University of Bonn (#21‐06‐30). Participants were recruited through personal contacts, flyers on the campus of the University of Bonn, mailing lists and online platforms. To ensure that all participation criteria were met, potential participants had to take part in an online pre‐screening and a laboratory screening before being invited to the fMRI session.

First, during the online pre‐screening (approximately 5 min), participants gave informed consent to take part in the online screening and were asked about the inclusion and exclusion criteria listed above. If they were deemed eligible for the study, they were invited to a laboratory screening.

Second, in the laboratory screening, participants provided informed consent for all further study procedures and were asked about the same information they had provided in the online screening. This included questions about the inclusion/exclusion criteria mentioned above as well as the MINI International Neuropsychiatric Interview (Sheehan et al., 1998; German translation by Ackenheil et al., 1999) to test for mental disorders and measurements of weight and height. Additionally, the UPPS‐P Impulsive Behavior Scale (Lynam et al., 2006; see Section 2.3) was assessed at this stage. Afterwards, participants completed all trials (27) of the delay condition and three trials of the date condition of the intertemporal choice task (see Section 2.3). Apart from demonstration purposes, delay condition data were also used to test for suitability to estimate the discount rate and to estimate the test–retest correlation across laboratory and fMRI sessions. The total duration of the laboratory screening was approximately 45 min. If participants passed the laboratory screening, a date was set for the fMRI session. The time period between laboratory screening and fMRI session varied between 0 and 19 days (*M* = 8 days).

Third, in the fMRI session at the MRI Core Facility of the Medical Faculty at the University Hospital of Bonn, female participants provided a urine sample which was tested for pregnancy (OneStep®, 10 miu/ml; none of the participants had to be excluded). Participants were once again informed about the fMRI procedures and criteria. Afterwards, they completed two different tasks in the fMRI scanner (the intertemporal choice task for this study as well as an eye movement task for a study published elsewhere [Schröder et al., 2023]). Afterwards, a structural T1 scan was obtained. The total duration of the fMRI session was approximately 90 min.

Participants were compensated with €30. Alternatively, psychology students from the University of Bonn could choose to be compensated with course credit. Additionally, all participants who had completed the fMRI session entered a lottery in which three winners were drawn. For these winners, one of their decisions in the intertemporal choice task during fMRI was randomly selected and paid out according to the choice made in the decision.

### 2.3 Materials

#### 2.3.1 Intertemporal choice task

The Monetary Choice Questionnaire (MCQ; Kirby et al., 1999) was used as intertemporal choice task. It comprises 27 items, asking participants to decide between a smaller‐immediate reward (SIR) and a larger‐later reward (LLR). In the current study, the MCQ was assessed in a German version. In the original MCQ, time attributes are given in intervals of days stating the waiting time (e.g., 24 Euro | 0 days vs. 35 Euro | 29 days; delay condition). We added a second set of 27 items with the time attributes in the format of dates (e.g., 24 Euro | November 24, 2021 [=today] vs. 35 Euro | December 23, 2021; date condition). Thus, each of the 27 original delay items has a corresponding date item. While the MCQ was also conducted in the laboratory (see Section 2.2), the following information only applies to the fMRI session.

The task was programmed using Experiment Builder software (SR Research Ltd., version 2.3.38) and displayed on a 32‐inch LCD monitor (NordicNeuroLab, 1920 × 1080px, height: 392.85 mm; width: 698.4 mm; refresh rate: 120 Hz). The monitor stood at the head end of the MR scanner, at a distance of approximately 190 cm from the participant's eyes. The task was presented to the participants using a first‐surface reflection mirror.

The MCQ was presented in blocks, each consisting of 27 trials of the respective condition (delay and date). The task consisted of a sequence of four blocks (2 per condition) in alternating order. The starting condition was counterbalanced between participants (i.e., A‐B‐A‐B or B‐A‐B‐A). Between blocks, participants had a break of 15 s before the next block began. The trial order within each block was randomised. SIR and LIR were presented simultaneously and side by side on the screen. Positions of SIR and LLR (left/right) were counterbalanced. Specifically, in half of the trials, the SIR was presented on the left side (LLR on the right side) of the screen and vice versa. The positions for the first delay block were randomly determined beforehand and were used for the corresponding items in the respective date block. These positions were also used for blocks 3 and 4 with reversed sides.

**FIGURE 1:**
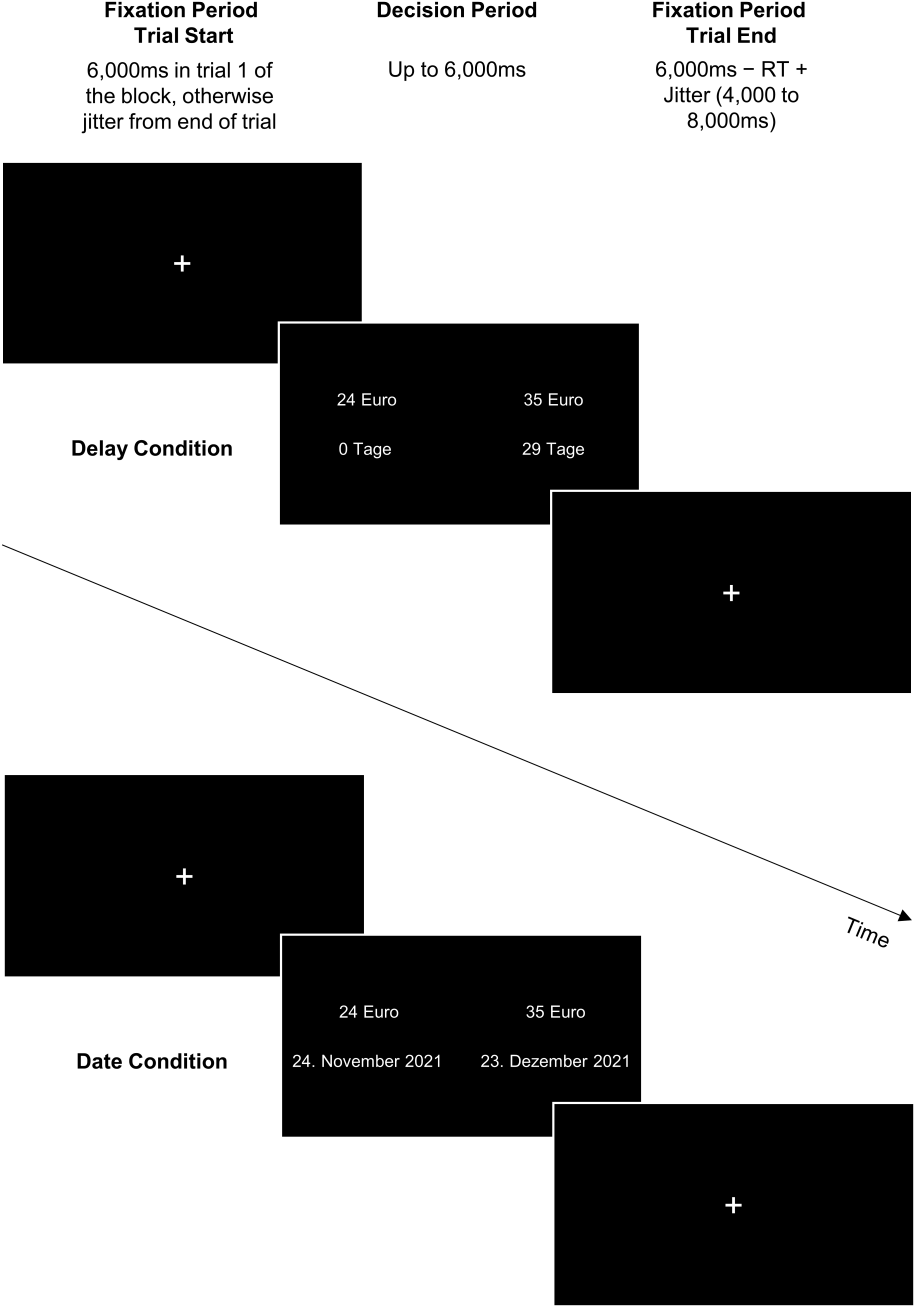
Illustration of the Intertemporal Choice Task. Each trial started with a fixation period. The duration was fixed at 6000 ms for the first trial and corresponded to the trial end fixation for the other trials. Afterwards, the decision screen (above: example of a delay trial; below: example of a date trial) was presented for 6000 ms during which a choice had to be made. Finally, a fixation cross was presented again. The duration of the latter stage was the sum of the time that would have been left during the decision screen (i.e., 6000 ms − reaction time [RT]) plus a jitter of 4000 ms to 8000 ms (drawn uniformly). All trials were presented in German (translation: Tage = days, Dezember = December). The font size was adjusted for better readability.

Before the start of the task, participants were instructed to press the buttons on the response grips (NNL, NordicNeuroLab) in their left and right hands with their index finger to choose the left or right option, respectively. Additionally, participants were reminded of the lottery and the current date. Each trial started with a fixation cross. The duration of the fixation period was 6000 ms in the first trial of a block or else depended on the decision time and jitter from the previous trial. Afterwards, the decision screen was presented. The decision screen showed the four choice attributes (two monetary amounts, two time attributes). Participants had to choose one of the options within 6000 ms; otherwise, the task proceeded to the next trial (without any error message). After the decision screen, a fixation cross was shown again (duration: 6000 ms—response time of the trial + inter‐trial interval [drawn uniformly from an interval between 4000 and 8000 ms]). Mean trial duration was approximately 12,000 ms. An example trial is illustrated in Figure 1.

#### 2.3.2 UPPS‐P Impulsive Behavior Scale

The UPPS‐P (Lynam et al., 2006) is a 59‐item questionnaire assessing five facets of trait impulsivity. It comprises the subscales positive urgency (tendency to react rashly after experiencing positive affect; 14 items), negative urgency (tendency to react rashly after experiencing negative affect; 12 items), sensation seeking (tendency to seek new and exciting experiences; 12 items), lack of premeditation (tendency to act without thinking; 11 items) and lack of perseverance (tendency to give up early on boring or hard tasks; 10 items). The UPPS‐P was completed in the laboratory. It was assessed in a German version (translation of the UPPS‐subscales from Schmidt et al., 2008, translation of the Positive Urgency subscale by the research group of the authors from the University of Bonn). Items were answered on a five‐point Likert scale (1 = strongly disagree, 2 = rather disagree, 3 = neutral, 4 = rather agree, 5 = strongly agree).

### 2.4 Data acquisition and preprocessing

#### 2.4.1 Choice behaviour and questionnaire

Choice behaviour was assessed using the MCQ. Trials with no answers within 6000 ms were excluded from behavioural analyses. The data revealed no unrealistic RTs (i.e., all RTs > 300 ms).

For the UPPS‐P subscales, sum scores were computed.

#### 2.4.2 Eye‐tracking

Eye movements were recorded using an MR‐compatible video‐based combined pupil and corneal reflection long‐range eye‐tracker EyeLink 1000 system (SR Research Ltd., Kanata, ON, Canada) at 1000 Hz sampling rate (no head tracking). The EyeLink 1000 has an end‐to‐end sample delay below 1.8 ms on average (SR Research Ltd., 2010). It measured horizontal and vertical gaze position data of the right eye in pixels on the screen, with (0,0) corresponding to the top‐left corner of the screen (note that pixels were transformed to degrees of visual angle from screen centre to indicate stimulus sizes and measurement uncertainty). The eye‐tracker was placed at the head end of the scanner bore, recording pupil and corneal reflection signals through the first‐surface reflection mirror through which the participant also saw the task. The participant's head was restrained by the head coil and cushions to minimise movement. Light was switched off during the task to ensure consistency of background lighting across participants. The eye‐tracker was calibrated and validated using a nine‐point horizontal‐vertical 3 × 3 grid (round white targets with a black dot in the middle, displayed in random order; coordinates: [960,540], [960,92], [960,988], [115,540], [1805,540], [115,92], [1805,92], [115,988], [1805,988]; visual angles were 9.19°, 4.90° and 10.38° of calibration stimuli positioned horizontally, vertically and diagonally from the screen centre, respectively). Calibration and validation followed the built‐in routines and settings provided by SR Research Ltd. We aimed to reach a validation classified as at least “fair” (i.e., worst point error should be below 2.0° and average error below 1.5°) and otherwise performed a new calibration. In the task, stimuli positions and sizes in degrees of visual angle were as follows: The fixation cross was positioned in the centre of the screen (0°, 0°), with a width of 1.11° and a height of 0.83°. The centres of reward and time attributes were always presented at a distance of 6.93° visual angle from the screen centre. The width of monetary amounts was 1.98°, and the width of times ranged from 1.75° (e.g., “0 Tage”) to 5.05° (e.g., “1 September 2022”). The height of all task stimuli was 0.52°. To determine whether a participant had looked at a specific attribute, we defined a priori areas of interest (AOIs) around the attribute centres (AOI width: 9.76°, AOI height: 2.46°).

Eye‐tracking events were preprocessed using the default settings of the EyeLink host software (version 4.56), unless specified otherwise. Data Viewer software (SR Research Ltd., version 4.2.1) was used to visually inspect data and generate data reports. All other preprocessing was done in R (version 4.2.2) using RStudio (version 2022.07.2). Since data inspection revealed major drift errors, we decided to recalibrate the data based on the fixation period before each trial. Specifically, for each trial, correction constants for *x*‐ and *y*‐coordinates were computed based on the deviations of gaze from the fixation cross in the last 4 s of the fixation period. These correction constants were then added to the original data of the decision period to correct for drift errors. Furthermore, to ensure that we only analysed eye‐tracking measures of trials with valid data, we excluded not only all trials in which participants did not answer in time (i.e., RTs as above) but also those in which data suggested that not all four AOIs were looked at. If this was the case for more than 20 trials in one of the conditions, we excluded the corresponding participant from eye‐tracking analyses, as this suggested either poor eye‐tracking data quality or a search strategy that excluded looking at specific AOIs. In total, *N* = 13 participants had to be excluded because they did not fulfil the criteria (trials with valid data: *M* = 25.78%, SD = 14.96%). Most of them (*N* = 9) had to be excluded because they did not look at the time attribute of the SIR in a sufficient number of trials (as this information was the same in all trials). Thus, data of *N* = 18 participants remained for the analyses (trials with valid data: *M* = 73.41%, SD = 16.39%). We note, however, that significantly fewer trials had to be excluded in the date (trials with valid data: *M* = 79.01%, SD = 18.42%) compared to the delay condition (trials with valid data: *M* = 67.80%, SD = 17.82%), *t*(17) = −3.07, *p* = .007, *g*_av_ = 0.61, which is probably due to the RT difference between conditions (see Section 3.1). Due to blinks, 1.08% (SD = 1.65%) of data in the decision period (per participant) were lost on average. Within‐participant comparisons showed that mean data loss due to blinks did not significantly differ between conditions (delay: *M* = 1.47%, SD = 2.74%; date: *M* = 0.82%, SD = 1.22%), *t*(17) = 1.16, *p* = .261, *g*_av_ = 0.32.

Empirical accuracy and precision of eye‐tracking measurements were analysed using the sample data of the first and last trial of the experiment. Specifically, we focused on the part of the fixation period earlier than 4 s before the decision period and selected the last fixation that ended before this time to ensure that data were independent of our recalibration procedure. Within each participant, we calculated the *x*‐ and *y*‐deviation in visual angle between the centre of the fixation cross and each sample of the fixation of the corresponding trial. Then, we computed the mean (for accuracy) and standard deviation (for precision) of the *x*‐ and *y*‐deviations for each sample within the fixation. We combined the measures for *x*‐ and *y*‐deviations using the Pythagorean theorem. Finally, we computed means of the combined measures to get indices for accuracy and precision. Results were as follows: For the first trial, mean accuracy was 3.68° (range: 0.54°–15.39°) and mean precision was 0.16° (range: 0.04°–0.36°). For the last trial, mean accuracy was 3.17° (range: 0.14°–5.71°) and mean precision was 0.25° (range: 0.07°–0.61°). While precision was relatively high, accuracy was relatively low. We suspect that this could be due to two reasons. First, the complicated setting (MR scanner), which also made the manual recalibration necessary, could have impeded data quality and the recalibration corrected for this only to a limited extent. Second, we speculate that participants might not have looked at the fixation cross throughout the whole fixation period, as it was relatively long. Thus, potential accuracy limitations should be considered when interpreting the eye‐tracking data.

#### 2.4.3 fMRI

Imaging data were collected with a 3 T MRI scanner (Siemens Magnetom Trio) using a 32‐channel head coil. During completion of the MCQ, a functional T2*‐weighted echo planar image (EPI) sequence was used to measure BOLD fMRI data (TR = 2500 ms, TE = 30 ms, flip angle = 90°, field of view = 192 mm, matrix size = 96 × 96, number of slices = 37, voxel size = 2 × 2 × 3 mm, interslice gap = 0.3 mm). Three‐dimensional T1‐weighted high‐resolution structural scans were obtained using Magnetization Prepared Rapid Acquisition with Gradient Echoes (MPRAGE) (TR = 1660 ms, TE = 2.54 ms, flip angle = 9°, field of view = 256 mm, matrix size = 320 × 320, number of slices = 208, voxel size = 0.8 × 0.8 × 0.8 mm).

BOLD data preprocessing was done in SPM12 using Matlab 2022b. Functional images were slice‐time corrected and then realigned to the first image of the time series using a least‐squares approach and a six‐parameter rigid body transformation. We segmented the individual structural T1‐weighted images into grey and white matter and cerebral spinal fluid and used the segmented image for co‐registration of anatomical and functional images. The co‐registered functional images were then normalised into standard Montreal Neurological Institute (MNI) space and smoothed using an 8 mm full width at half maximum (FWHM) Gaussian kernel.

### 2.5 Statistical analyses

All behavioural analyses were conducted in R (version 4.2.2) using RStudio (version 2022.07.2) and Rstan (version 2.26.1). BOLD analyses were done in SPM12 using Matlab 2022b.

#### 2.5.1 Estimation of discount rates

Log‐transformed discount rates were estimated using hierarchical Bayesian modelling in Rstan. Specifically, for choice data from the laboratory, we fitted a computational model assuming a hyperbolic function to get initial estimates of the individual discount function. For choice data from the fMRI task, we fitted a computational model to estimate discount rates, assuming each a hyperbolic, an exponential and a quasi‐hyperbolic function. Using leave‐one‐out cross‐validation to compare model fits (LOO‐CV; Vehtari et al., 2017),^1^ the hyperbolic function appeared to fit the data best. While differences between predictive accuracies of the different models were small, we nevertheless decided to use discount rates estimated based on the hyperbolic function, as this is also in line with previous studies (Kable & Glimcher, 2007; Keidel, Murawski, & Ettinger, 2023; Peters & Büchel, 2010). We estimated separate discount rates for the delay and date conditions. To get an estimate of the date/delay effect (date premium), we subtracted the estimated log‐transformed discount rates from each other (date minus delay). That is, more negative values reflect a higher date/delay effect.

#### 2.5.2 Behavioural analyses

First, we computed the test–retest reliability of temporal discounting by computing the Pearson correlation between log‐transformed discount rates in the delay condition in the laboratory and fMRI. Then, to analyse whether the date/delay effect was replicated, we inspected the 95% highest density intervals (HDIs) of the estimated log‐transformed discount rates from the fMRI task and used the Bayesian Estimation Supersedes the *t*‐test (BEST) approach (Bååth, 2014; Kruschke, 2013) to estimate the probability with which the paired difference of the point estimates of discount rates, that is, the date premium, differed from zero. Additionally, we compared the point estimates of the log‐transformed discount rates in the delay and date conditions using a paired‐sample *t*‐test. We also used paired *t*‐tests to examine whether reaction times (RTs) and log‐transformed RTs differed between conditions.

Next, we carried out eye‐tracking data analyses similar to those reported in Keidel, Murawski, and Ettinger (2023). We computed means for saccade frequencies within different AOIs, saccade frequencies between different AOIs (based on fixation data; a change of fixated AOI was considered a saccade) and cumulative fixation durations within different AOIs. Moreover, we created summary measures for saccade direction (vertical/horizontal) and fixation focus (reward/time) for each condition. Specifically, for saccade direction, we computed the Payne index (Payne, 1976), which represents the ratio of the difference of vertical and horizontal saccades relative to their sum. Values range from −1 to 1, with values higher than zero reflecting more vertical eye movements (i.e., a more integrative strategy) and values below zero reflecting more horizontal eye movements (i.e., a more comparative strategy). For fixation focus, we computed a similar index based on the fixation durations ranging between −1 and 1 (Amasino et al., 2019, formula 4), where values higher than zero reflected a relative reward focus and values below zero reflected a relative time focus. Given that we analysed eye‐tracking data from 18 participants only (see Section 2.4.2) and wanted to reduce the influence of outliers, we excluded all individual data of variables used in the analyses that were above or below 2.5 standard deviations of the sample mean. We then used two paired *t*‐tests to compare the number of saccades within AOIs and between AOIs (horizontal, vertical, diagonal) between the delay and date conditions. To investigate differences in the number of vertical vs. horizontal saccades between AOIs in the two conditions (delay vs. date), we conducted a paired *t*‐test comparing the Payne indices. To examine this in more detail, we examined differences between conditions in the number of all possible types of horizontal and vertical saccades (horizontal between rewards vs. horizontal between times vs. vertical between SIR attributes vs. vertical between LLR attributes) using further paired *t*‐tests. We then compared fixation durations on attribute types (reward vs. time) in the two conditions (delay vs. date) using a 2 × 2 repeated‐measures ANOVA. Effects in this and the following ANOVA were further examined using Bonferroni‐Holm corrected post hoc *t*‐tests. To analyse whether the effects were the same for each option, we added an additional factor for the option (SIR vs. LLR) and conducted a 2 × 2 × 2 repeated‐measures ANOVA. Then, to see whether the differences we found on the within‐subject level were also predictive of the between‐subject magnitude of the date/delay effect, we ran a linear regression analysis of the date/delay effect on difference measures (date minus delay) of saccade direction (i.e., Payne index) and of fixation focus measures. Similarly, we calculated Pearson correlations between temporal discounting and eye‐tracking measures (as in Keidel, Murawski, & Ettinger, 2023). Finally, to see whether differences in attribute values interacted with condition to predict choice behaviour, we ran a linear mixed model with choice as dependent variable (0 = SIR;1 = LLR). Condition (effect coded: −1 = delay, 1 = date), difference in reward (Δreward) and difference in time (Δtime), and their interactions were modelled as fixed effects. We included participant random effects on the intercept.

#### 2.5.3 fMRI analyses

The preprocessed BOLD data were used to estimate a general linear model (GLM) at the first level. Conditions were modelled as separate regressors by convolving variable boxcar functions (trial onset until reaction) with the hemodynamic response function for each trial. We added the sum of the SVs of both options of a trial as parametric modulator (Reeck et al., 2021). Specifically, for each trial, the SV of each option was computed using the hyperbolic discount function as follows: SV = A/(1 + *k*D), where SV represents the subjective value of the chosen option, A is the objective value of the reward, *k* is the discount rate for the respective condition and D is the delay (Mazur, 1987). Note that for the SIR, SV = A. The sum of SVs was demeaned and added to the respective condition. In SPM, the parametric regressor is automatically orthogonalised with respect to the unmodulated regressor (Mumford et al., 2015). Thus, in total, four regressors of interest were included in the GLM: delay trial, modulation of the delay trial by SV, date trial, modulation of the date trial by SV. The six motion parameters from realignment were added as regressors of no interest. Fixations and pauses between blocks (note that data acquisition continued through pauses) were not modelled and served as implicit baseline. Slow‐signal drifts were removed using a 128‐s high‐pass filter.

We computed first‐level contrasts by comparing the delay and date regressors to an implicit baseline (delay > baseline, date > baseline) and against each other (delay > date, date > delay). The first‐level contrasts were then analysed on the second level (whole‐brain) using random‐effect one‐sample *t*‐tests. We applied a familywise error rate (FWE) corrected voxel‐related threshold of *p* < .05 and a minimum cluster size of 10 voxels for the contrasts against baseline and a voxel‐related threshold of *p* < .001 and an FWE‐corrected cluster threshold, following the SPM procedure, for the comparison of conditions and all following analyses.

Next, we analysed the parametric modulation effects of SV. Following a whole‐brain analysis of SV differences between conditions, we used a region of interest (ROI) approach. Specifically, given that the SV signal is usually found in bilateral vS and bilateral ventro‐medial prefrontal cortex (vmPFC) (Bartra et al., 2013; Frost & McNaughton, 2017; Kable & Glimcher, 2007; Moreira et al., 2016), we defined ROIs according to the meta‐analysis by Bartra et al. (2013) using masks from their fig. 9 (https://www.sas.upenn.edu/~mcguirej/meta-analysis.html). We compared parametric modulation effects of the sum of SVs in vS and vmPFC against baseline and between conditions. In additional analyses, we replaced the sum of SVs with the SV of the chosen option, the difference of SVs (LLR − SIR) and the ratio of SVs (LLR/SIR) as parametric modulators. To examine SV effects more precisely, we additionally computed two models in which differences (or sums) of objective rewards and objective timings of the options were included as parametric modulators. As objective reward and timing differences are correlated in the MCQ, we used the automated orthogonalisation in SPM and set up two separate models in which either reward or timing was entered first and the other entered second to obtain variances specific to the variable entered second (Mumford et al., 2015).

We then analysed individual differences in the date/delay effect by adding discount rates in the two conditions and the date/delay effect as second‐level covariates to the corresponding contrast images of the original model. Specifically, we examined in which brain regions the discount rates in the two conditions were associated with the delay > baseline and date > baseline contrasts and in which brain regions the magnitude of the date/delay effect was associated with the date > delay contrast. Similarly, to explore potential associations with personality, we also used the UPPS‐P subscale scores as covariates in the date > delay contrast.

Finally, we used Pearson's correlations to explore whether contrast estimates of significant clusters in the date > delay contrast as well as the date/delay effect covariate effects in this contrast were associated with the eye‐tracking difference measures.

## 3 RESULTS

### 3.1 Behavioural analyses

The test–retest reliability of the log‐transformed discount rate estimates in the standard delay condition between the laboratory and fMRI sessions was *r*(29) = .70. When excluding one outlier, this correlation increased to *r*(28) = .81.

The 95% HDIs of the posterior distributions of the log‐transformed discount rates were (−6.19; −5.28) in the date and (−5.89; −4.75) in the delay condition. The mean paired difference of their point estimates was −0.43 with a 95% HDI of (−0.66; −0.20) and had a probability of more than 99.9% of being less than zero, replicating the date/delay effect. Similarly, a paired *t*‐test showed reduced temporal discounting in the date (*M* = −5.72, SD = 1.17) compared to the delay condition (*M* = −5.27, SD = 1.55), *t*(30) = −3.96, *p* < .001, *g*_av_ = 0.33. Descriptive values for each condition revealed that the majority of participants showed the date/delay effect in the expected direction but that this effect varied in magnitude between participants (Supplement A).

Moreover, we found that participants had higher RTs in the date (*M* = 2.59 s, SD = 0.66 s) compared to the delay condition (*M* = 2.26 s, SD = 0.55 s), *t*(30) = 7.22, *p* < .001, *g*_av_ = 0.54. Given that RTs within participants were characteristically skewed to the right (Whelan, 2008), with an average within‐participant skewness of 0.94 (SE = 0.08), we tested whether this effect was robust by log‐transforming the RTs and comparing their means. Results of the previous analysis were confirmed, *t*(30) = 7.31, *p* < .001, *g*_av_ = 0.53.

With regard to eye‐tracking, we found that participants made more saccades within AOIs in the date (*M* = 3.48, SD = 1.38) than in the delay condition (*M* = 2.45, SD = 1.01), *t*(17) = 6.87, *p* < .001, *g*_av_ = 0.84, but the number of saccades between AOIs did not differ significantly (delay: *M* = 4.66, SD = 1.10; date: *M* = 4.81, SD = 1.00), *t*(16) = −0.74, *p* = .470, *g*_av_ = 0.21.

**FIGURE 2:**
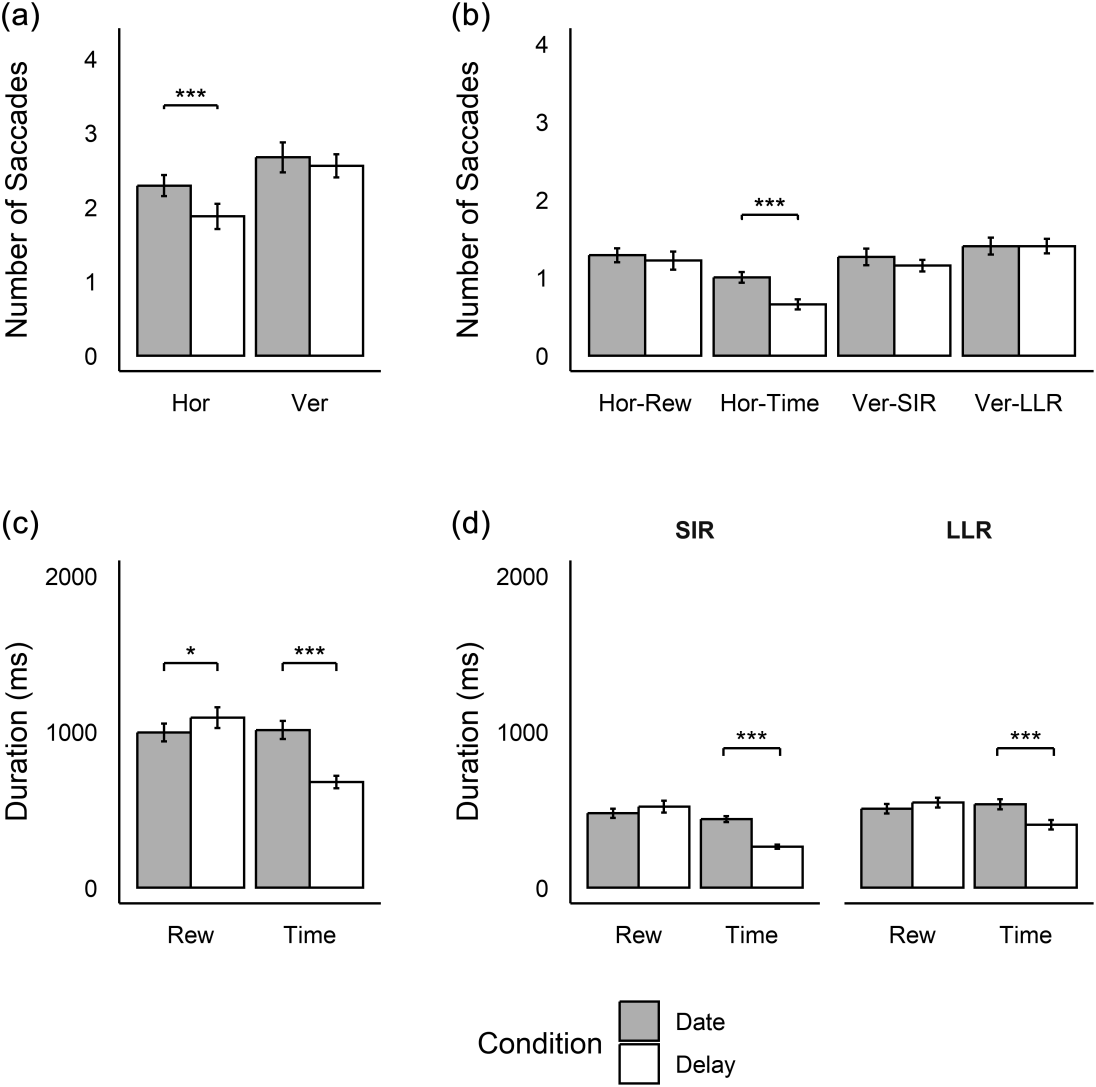
Number of Saccades Between Areas of Interest (AOIs) and Duration of Fixations Within AOIs. (a) represents all horizontal (Hor) and vertical (Ver) saccades between AOIs, and (b) represents saccades separated by attribute‐wise (Rew = reward), horizontal saccades and option‐wise (SIR, smaller‐immediate reward; LLR, larger‐later reward), vertical saccades. (c) represents the fixation duration on reward and time AOIs, summarised over options, and (d) represents fixation durations on reward and time AOIs, separated by options. Error bars represent standard errors of the mean. Asterisks are only depicted for post hoc *t*‐tests within the same category (i.e., bars next to each other). *p_adj_ < .05, ***p_adj_ < .001 (Bonferroni‐Holm corrected for multiple comparisons).

Focusing on differences between conditions in the frequency of vertical vs. horizontal saccades between AOIs (Figure 2a), a paired *t*‐test revealed that the Payne index, that is, the proportion of vertical relative to horizontal saccades, was lower in the date than in the delay condition, *t*(17) = −3.09, *p* = .007, *g*_av_ = 0.41. Separate Bonferroni‐Holm corrected *t*‐tests showed that participants made significantly more horizontal saccades in the date than in the delay condition, *t*(17) = 5.13, *p*_adj_ < .001, *g*_av_ = 0.61, while the frequency of vertical saccades did not significantly differ between conditions, *t*(17) = 0.98, *p*_adj_ = .341, *g*_av_ = 0.15. Additional Bonferroni‐Holm corrected *t*‐tests comparing differences between conditions for all possible types of saccade direction (Figure 2b) showed that the difference in horizontal saccades could be traced back to the higher number of saccades between time attributes in the date compared to the delay condition, *t*(17) = 7.73, *p*_adj_ < .001, *g*_av_ = 1.20 (all other *p*_adj_ > .05).

To analyse fixation durations within AOIs, we conducted a 2 × 2 repeated‐measures ANOVA, using condition (delay vs. date) and attribute type (reward vs. time) as factors (Figure 2c). We found a significant interaction effect, *F*(1,16) = 65.02, *p* < .001, $\eta_{p}^{2}$ = 0.80, and main effects of condition, *F*(1,16) = 20.69, *p* < .001, $\eta_{p}^{2}$ = 0.56, and attribute type, *F*(1,16) = 25.11, *p* < .001, $\eta_{p}^{2}$ = 0.61. Post hoc *t*‐tests revealed that participants fixated time attributes significantly longer in the date than in the delay condition, *t*(16) = 8.75, *p*_adj_ < .001, *g*_av_ = 1.59, and reward attributes significantly shorter in the date than the delay condition, *t*(16) = −2.58, *p*_adj_ = .020, *g*_av_ = 0.36. To distinguish between all four AOIs, we conducted a 2 × 2 × 2 repeated‐measures ANOVA that additionally included option (SIR vs. LLR) as a factor (Figure 2d). Most importantly, we found a significant two‐way interaction between condition and attribute type, *F*(1,15) = 103.74, *p* < .001, $\eta_{p}^{2}$ = 0.87 (complete ANOVA results in Supplement B1). Post hoc *t*‐tests indicated that this effect emerged for both SIR, *t*(15) = 10.50, *p*_adj_ < .001, *g*_av_ = 2.56, and LLR, *t*(15) = 6.89, *p*_adj_ < .001, *g*_av_ = 1.03 (other post hoc *t*‐test comparing fixation durations for attribute types in the two conditions: *p*_adj_ > .05). Thus, we found that participants fixated time attributes of both SIR and LLR longer in the date than in the delay condition, while there was no significant difference for the reward attributes. Similar results emerged when analysing the number of saccades and fixations within AOIs (Supplement B2 and B3).

On a between‐subject level, a linear regression showed that the date/delay effect was not significantly predicted by difference measures of the proportion of vertical (vs. horizontal) saccades, *β* = 0.02, *t*(14) = 0.06, *p* = .950, and the fixation duration on rewards (vs. times), *β* = 0.21, *t*(14) = 0.81, *p* = .431 (model *R* ^2^ = .05, *p* = .725). Pearson correlations of temporal discounting and eye‐tracking measures (which reflect in part the same calculations as in tab. 4 of Keidel, Murawski, & Ettinger, 2023) can be found in Supplement C1 and C2 and Pearson's correlations of temporal discounting and UPPS‐P variables (not yielding any significant effects) in Supplement D. Generally, all but one of the significant associations in Supplement C1 are in the same direction as in Keidel, Murawski, and Ettinger (2023).

Finally, to analyse whether differences in reward or time values (or both) interacted with condition to predict choice behaviour, we ran a mixed‐effect model predicting choice behaviour (0 = SIR; 1 = LLR) by condition (effect coded: −1 for delay, 1 for date), absolute differences in reward (Δreward) and time (Δtime) and their interactions. While we found that, expectedly, the date condition, *β* = 0.44, *p* < .001, a higher Δreward, *β* = 1.86, *p* < .001, and a lower Δtime, *β* = −2.94, *p* < .001, significantly predicted a higher probability of choosing the LLR, we did not find any significant interactions (Supplement E).

### 3.2 fMRI analyses

#### 3.2.1 BOLD activation in delay and date conditions

**FIGURE 3:**
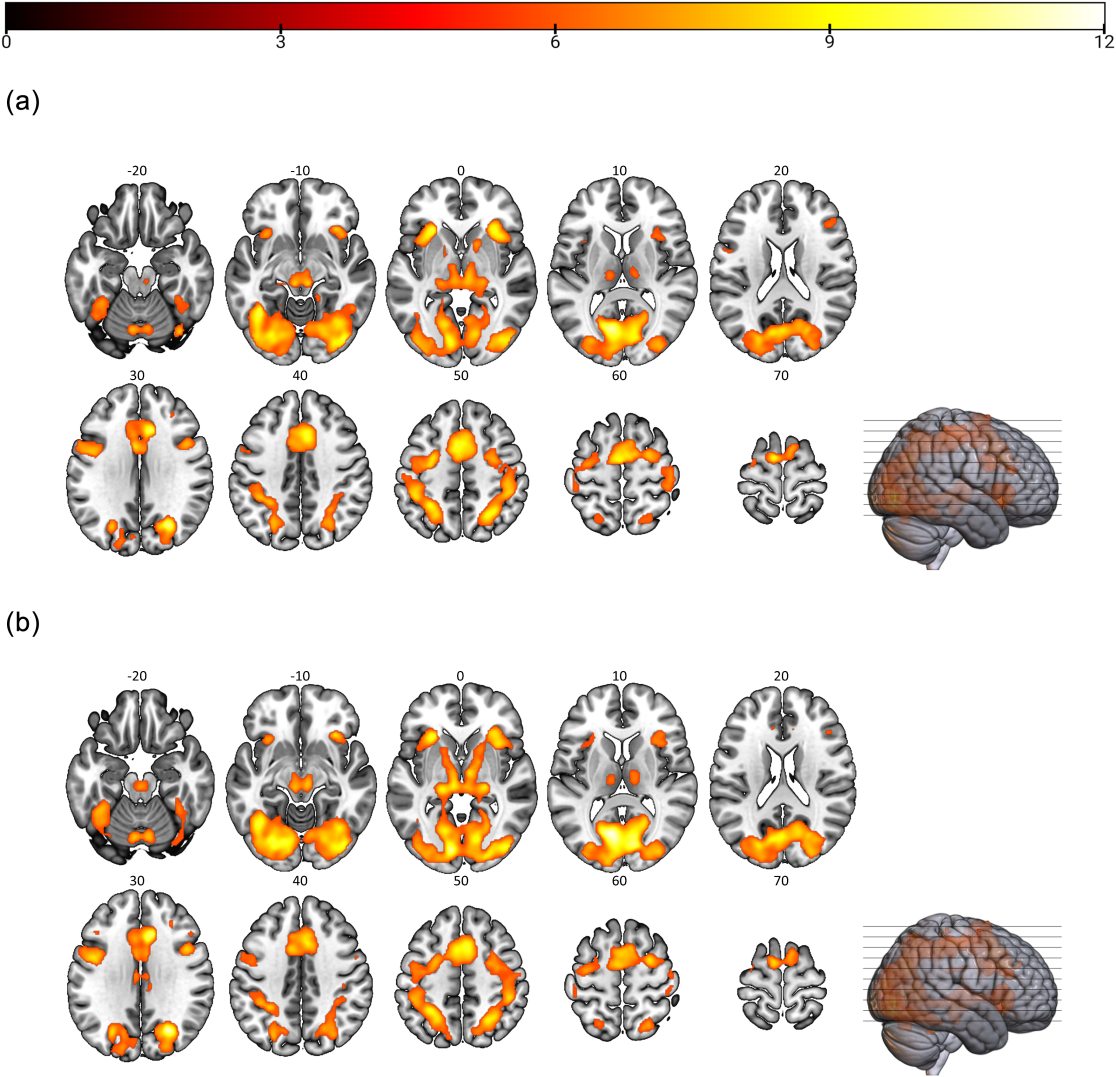
Blood‐oxygen level‐dependent (BOLD) signal during intertemporal choices. Depicted are significant areas from one‐sample *t* contrasts (a) delay > baseline and (b) date > baseline with an FWE‐corrected voxel‐related threshold (*p* < .05) and a minimum cluster size of 10 voxels. Intensities of colours reflect the heights of *t*‐values, as shown in the colour bar. Number labels refer to Montreal Neurological Institute (MNI) *z*‐coordinates.

When compared separately to baseline, the delay and date conditions activated largely similar regions (Figure 3, Supplement F1 and F2). Specifically, compared to baseline, both conditions led to higher activations in occipital cortex (including V1, V3), anterior/mid‐cingulate cortex (ACC, MCC), insula and inferior frontal gyrus. The delay condition also activated small clusters in caudate and putamen.

**FIGURE 4:**
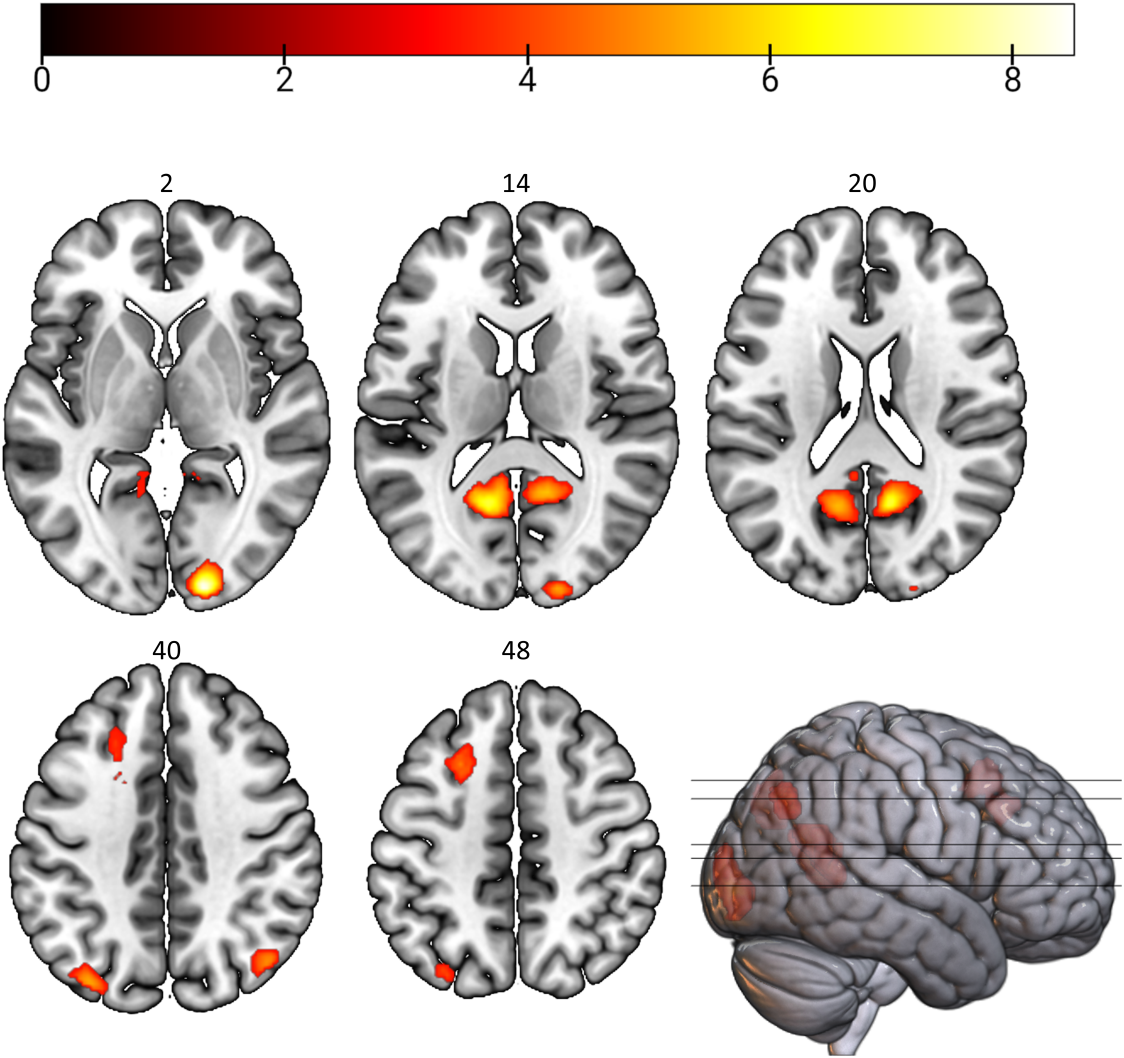
Blood‐oxygen level‐dependent (BOLD) signal in Date > Delay. Depicted are significant areas from a one‐sample *t* contrast date > delay with a voxel‐related threshold of *p* < .001 and an FWE‐corrected cluster threshold. Intensity of colours reflects the heights of *t*‐values, as shown in the colour bar. For the delay > date contrast, no significant clusters emerged. Number labels refer to Montreal Neurological Institute (MNI) *z*‐coordinates.

Comparing the two conditions, we did not find any significant differences for the contrast delay > date. However, in the contrast date > delay, we found higher activation in visual areas, left posterior cingulate cortex (PCC), right precuneus, left middle/superior frontal gyrus (MFG/SFG) and bilateral angular gyrus (Figure 4, Table 1).

**TABLE 1:** Blood‐oxygen level‐dependent (BOLD) signal in Date > Delay.

Anatomical label (functional label)	Cluster size	*t*‐value	MNI coordinates		
			*x*	*y*	*z*
L Calcarine Gyrus	624	6.93	−12	−58	14
L PCC		4.77	−6	−48	16
R Precuneus	522	7.01	10	−60	20
R Precuneus		5.69	14	−54	12
R Lingual Gyrus		4.17	8	−46	6
R Calcarine Gyrus (V1)	505	8.49	16	−96	2
R Lingual Gyrus (V3)		8.48	18	−94	−2
L Middle Occipital Gyrus	365	5.32	−34	−78	40
L Middle Occipital Gyrus (IPL)		5.17	−40	−78	32
L Middle Occipital Gyrus (IPL)		5.12	−42	−76	34
L Angular Gyrus (IPL)		5.00	−46	−74	32
L Middle Frontal Gyrus	350	4.96	−24	16	48
L Middle Frontal Gyrus		4.84	−28	18	52
L Middle Frontal Gyrus		4.67	−24	18	54
N/A^a^		4.52	−22	22	36
N/A^a^		4.51	−22	26	34
N/A^a^		4.39	−22	10	46
L Superior Frontal Gyrus		4.23	−22	22	46
L Middle Frontal Gyrus		3.70	−18	26	42
R Angular Gyrus (IPL)	164	4.99	44	−70	40

*Note*: The Anatomy Toolbox (Eickhoff et al., 2005) was used to infer anatomical labels. In case of an unknown area (N/A), we additionally specified the region in the footnote using the Neuromorphometrics atlas in SPM. Cluster size is given in number of voxels. MNI = Montreal Neurological Institute.

^a^ L Cerebral White Matter.

#### 3.2.2 Analysis of the subjective value signal

First, a whole‐brain analysis of the SV signal did not yield any significant activation differences between conditions. Next, in the ROI analyses focusing on vS and vmPFC, we found that a higher sum of SVs of SIR and LLR was associated with stronger activation in vS in the delay condition, *t*(30) = 2.60, *p* = .014, *d* = 0.47, but not in the date condition, *t*(30) = −0.42, *p* = .674, *d* = 0.08. Comparing the two conditions, the association in the delay condition was significantly higher than in the date condition, *t*(30) = 2.18, *p* = .037, *g*_av_ = 0.57. We did not find any significant effects for the vmPFC (all *p* > .05). While similar results emerged when using the SV of the chosen option as regressor, the correlations were not significant when using the difference or the ratio of SVs of SIR and LLR (Supplement G1). Consistently, however, whenever significant effects emerged, SV only correlated with neural activity in the delay but not the date condition, suggesting a more robust SV signal in the delay condition. Models in which both objective rewards and timings were included as parametric modulators showed that both higher reward differences and higher time differences were associated with higher vS activity in the delay (reward differences: *t*(30) = 2.97, *p* = .006, *d* = 0.53; time differences: *t*(30) = 2.27, *p* = .030, *d* = 0.41) but not in the date condition (reward differences: *t*(30) = 0.70, *p* = .487, *d* = 0.13; time differences: *t*(30) = 0.80, *p* = .431, *d* = 0.14), although the differences between the two conditions were not significant (*p* > .05). No significant effects emerged for the vmPFC (all *p* > .05). Similar results emerged when using reward and timing sums, except that (i) the difference in the association of reward sums and vS activity was significantly higher in the delay than in the date condition, and (ii) the objective time effect in the delay condition was not significant (Supplement G2). In sum, we found an SV signal as well as objective reward and (potentially) time signals in the vS in the delay but not in the date condition.

#### 3.2.3 Covariates of activation differences in date and delay conditions

**FIGURE 5:**
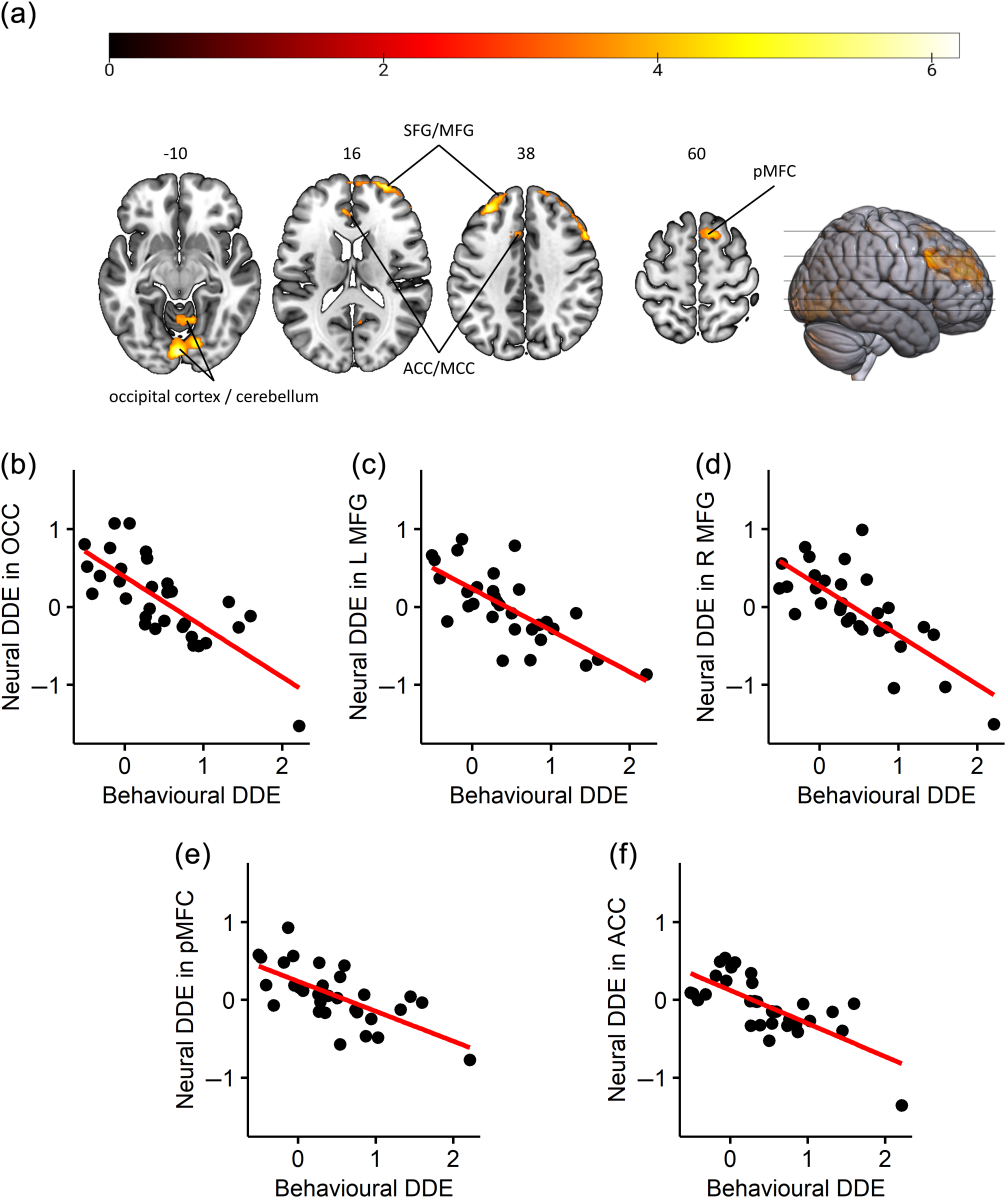
Associations Between the Behavioural Date/Delay Effect and the Neural Date/Delay Effect. The behavioural date/delay effect (DDE), that is, ln(*k*)_Date_–ln(*k*)_Delay_, was used as covariate for the date > delay contrast (neural DDE). (a) represents significant areas at a voxel‐related threshold of *p* < .001 and an FWE‐corrected cluster threshold. Intensity of colours reflects the heights of *t*‐values, as shown in the colour bar. Upper labels refer to Montreal Neurological Institute (MNI) *z*‐coordinates. As the behavioural DDE was initially negatively coded (date minus delay), that is, lower values represent a stronger behavioural DDE, activity in these areas differed more strongly in people showing a smaller (or opposite) behavioural DDE. (b)–(f) illustrate the direction of the DDE‐dependent signal changes in significant clusters. For better interpretation, the *x*‐axis was recoded so that higher values represent a higher behavioural DDE (i.e., ln(*k*)_Delay_–ln(*k*)_Date_). ACC, anterior cingulate cortex; L, left; MCC, medial cingulate cortex; MFG, middle frontal gyrus; OCC, occipital regions; pMFC, posterior medial frontal cortex; R, right; SFG, superior frontal gyrus.

We explored whether individual discount rates covaried with activation in the contrasts delay > baseline and date > baseline. Using the individual discount rates in each condition as covariate, we did not find any significant correlations. However, using the date/delay effect as covariate in the date > delay contrast, we found that a lower date/delay effect correlated with higher activity differences in bilateral occipital areas and cerebellum, right SFG, bilateral MFG, bilateral posterior medial frontal cortex (pMFC) and left ACC and MCC (Figure 5, Supplement H).^2^ That is, people who had a less pronounced behavioural date/delay effect or the opposite effect (i.e., higher discounting in the date compared to the delay condition), showed activity that differed more strongly between conditions in brain regions associated with visual information processing, attention and eye movements as well as cognitive control.

Using the UPPS‐P scores as covariates, we did not find any significant clusters in the date > delay contrast. Thus, we did not find any significant correlations between the neural date/delay effect and the five trait impulsivity constructs assessed with the UPPS‐P.

### 3.3 Explorative correlations of Eye‐tracking and fMRI analyses

In the eye‐tracking sample, we examined the between‐subject relationships of significant clusters in the contrast date > delay and the covariate effects of the date/delay effect on this contrast with difference measures of the proportion of vertical (vs. horizontal) saccades and the fixation duration on rewards (vs. times) (Supplement I1 and I2). First, for the date > delay contrast, we found a significant negative correlation between the occipital cortex cluster and the duration of fixations on rewards (vs. times), *r*(16) = −.50, *p* = .034. That is, higher fixation durations on time attributes were associated with stronger activation in occipital cortex in the date compared to the delay condition, likely representing the higher number of symbols in dates compared to delay units. Second, for the effects of the date/delay effect as covariate in the date > delay contrast, we did not find any significant correlations.

## 4 DISCUSSION

We aimed to elucidate the mechanisms of the date/delay effect in intertemporal choice using eye‐tracking and fMRI. Results showed that the date condition led to lower discount rates, replicating the date/delay effect (Dshemuchadse et al., 2013; Jiang & Dai, 2021; Keidel, Murawski, & Ettinger, 2023; Keidel, Murawski, Pantelis, & Ettinger, 2023; LeBoeuf, 2006; Naudé et al., 2018; Read et al., 2005). We also found higher RTs in the date compared to the delay condition. Furthermore, eye‐tracking revealed a higher proportion of saccades between time attributes and higher fixation durations on time attributes in the date compared to the delay condition, also replicating previous results (Keidel, Murawski, & Ettinger, 2023). Importantly, we observed greater activation in visual areas, angular gyrus, precuneus, and MFG/SFG in the date than in the delay condition. Furthermore, we detected a higher SV signal in the vS in the delay compared to the date condition. Lastly, we found significant correlations between lower behavioural date/delay effects (or opposite effects) and higher activity differences in bilateral occipital areas (including cerebellum), right SFG, bilateral MFG, pMFC, and ACC/MCC in the date > delay contrast.

### 4.1 Mechanisms of the date/delay effect

With regard to the cognitive mechanisms of the date/delay effect, our results suggest that differences in time information processing play a role, as suggested by the differential time estimation hypothesis (Read et al., 2005). Specifically, replicating our previous study (Keidel, Murawski, & Ettinger, 2023), we found that people spent more time reading/processing and comparing dates than delay units, suggesting stronger consideration of time information (at least in overt attention). While the former finding alone could also be caused by different reading requirements in the two conditions, the current study emphasised the role of time processing by revealing higher activity in precuneus/PCC and angular gyrus (lateral parietal cortex) in the date than in the delay condition. These regions are associated with episodic thinking (Schacter et al., 2007) and as such are important for the memory‐driven system specified by Frost and McNaughton (2017).

Interestingly, the regions identified in our study are similar to those found in a study examining the neural effects of episodic future thinking tags (tag effect) on intertemporal choices (Peters & Büchel, 2010; Sasse et al., 2015), which also have been shown to reduce temporal discounting (Rung & Madden, 2018; H. Scholten et al., 2019). Following Peters and Büchel, it would be possible that “similar mechanisms may underlie the decrease in discounting reported for date‐based processing relative to that of delay‐based processing (Read et al., 2005) and the tag effect” (Peters & Büchel, 2010, p. 144), that is, a stimulation of visual imagery through framing cues. Alternatively, activity in our study could also reflect higher specificity of the date cues and the resulting possibility for dates to act as a stimulus to trigger memories. In both cases, activity in episodic thinking regions could indicate higher personal relevance and lower psychological distance and, thus, would result in a subjectively closer temporal proximity of the LLR and as such represent a neural equivalent of the lower‐level construal of dates compared to delay units suggested by Construal Level Theory (Trope & Liberman, 2003). Indeed, previous studies on Construal Level Theory emphasised the role of precuneus/PCC for lower‐level construals (Stillman et al., 2017; Tamir & Mitchell, 2011), also within intertemporal choices (Lee et al., 2022). Thus, the date/delay effect can be explained by lower estimates of delay in the date compared to the delay condition—either through relatively more vivid anticipation of future rewards or through memory contents activated by higher specificity of dates. Future studies should therefore consider including subjective time estimate tasks in their designs, as time perception has been shown to influence intertemporal decision‐making on behavioural (Zauberman et al., 2009) and neural levels (Cooper et al., 2013). Also, asking participants whether they experienced more vivid imagery or specificity in the date condition (Peters & Büchel, 2010) would be useful to pinpoint the cause of differential time estimation.

An additional explanation to a differential time estimation could be that, although reward attributes are not processed more intensively in the date compared to the delay condition, they may be weighted more heavily than time attributes in the ultimate decision process (Keidel, Murawski, & Ettinger, 2023; LeBoeuf, 2006), potentially mediated through more vivid anticipation of a delayed reward. However, in this study, we did not find a significant association of differences in fixation durations on rewards between the two conditions and the magnitude of the date/delay effect, nor an interaction between condition and Δreward in our linear mixed‐model (unlike Keidel, Murawski, & Ettinger, 2023), nor any neural correlates suggesting a stronger reward/SV signal in the date condition. Conversely, we found a higher SV signal in the vS in the delay condition, which could reflect higher valuation of the SIR, the LLR or both. Thus, these findings make a higher valuation of rewards in the date condition appear very unlikely. However, as the previous study found Δreward to be more important in the date than in the delay condition in a larger sample (Keidel, Murawski, & Ettinger, 2023), it may be possible that a relatively higher reward weighting assigned to the LLR only occurs in a later, cognitively self‐controlled choice stage of the decision‐making process (Liu et al., 2012). As different stages of decision‐making were not separated in our design, it would be useful in future to separate valuation and choice stages (Liu et al., 2012) to examine which differences occur in which parts of the decision process.

Lastly, according to the choice strategy hypothesis, participants either need to perform complex transformations (i.e., calculating delay units from dates) to use the same strategy in the date as in the delay condition or they need to adapt their strategy (Read et al., 2005). The higher activity in left MFG/SFG in the date > delay contrast might initially suggest more complex transformations in the date than the delay condition because these regions are involved in arithmetic operations (Arsalidou & Taylor, 2011; Fehr et al., 2007). However, the interindividual effects in more widespread prefrontal areas (including MFG/SFG) suggest that this effect is higher when the date/delay effect is lower. That is, higher activity in the SFG/MFG, pMFC and ACC/MCC in the date compared to the delay condition is associated with a smaller behavioural date/delay effect (or even an opposite effect). Given that these regions are involved in cognitive control (Niendam et al., 2012; Ridderinkhof et al., 2004; Wang et al., 2010; for neural intertemporal choice models involving self‐control, see Figner et al., 2010; Liu et al., 2012) and—for SFG/MFG—in arithmetic operations (Arsalidou & Taylor, 2011; Fehr et al., 2007), this could suggest that people with a smaller behavioural date/delay effect rely on the same strategy in the two conditions but engage cognitive control or arithmetic processes more strongly, for example by transforming dates to delay units. On the other hand, people with a higher behavioural date/delay effect may calculate less and use a different, potentially more intuitive decision‐making strategy to adapt to the date condition (e.g., they may rely more on episodic processes), supporting the choice strategy hypothesis. This strategy could be reflected by the higher total proportion of saccades between time attributes in the date than in the delay condition, replicating previous results (Keidel, Murawski, & Ettinger, 2023). We note, however, that these interpretations are rather speculative. Specifically, it should be noted that the direction in the association between activation of cognitive control regions and the size of the date/delay effect is not clear (i.e., it could also be interpreted that persons with a higher date/delay effect show higher activity in cognitive control regions for the delay > date contrast, reflecting more arithmetic operations in the delay than in the date condition). Moreover, our study did not find a significant association between the difference in strategy use and the date/delay effect. More generally, we note that our study was not sufficiently powered to draw robust conclusions about between‐participant associations regarding the eye‐tracking measures. Nevertheless, while the eye‐tracking results replicate previous explorative findings (Keidel, Murawski, & Ettinger, 2023), our fMRI findings provide tentative evidence for the choice‐strategy hypothesis on the neural level. Future studies could examine eye movements in larger samples using an intertemporal choice task with varying times of smaller‐sooner rewards to manipulate the difficulty of estimating time differences and examine the effects on reward weighting (and to reduce potentially necessary exclusions, see Section 4.2).

Finally, we note that we did not find correlations of the date/delay effect with trait impulsivity according to the UPPS‐P scales, neither on the behavioural nor on the neural level. Even if the power to detect such associations was limited, this suggests that differences in the date/delay effect (and in prefrontal activity) do not strongly reflect trait impulsivity differences but rather a specific task‐dependent effect, for example, interindividual differences in the tendency to use comparative strategies and to calculate time differences. Nevertheless, interindividual differences such as intelligence or personality traits could be important, not least because they covary with temporal discounting itself (Keidel et al., 2021). Large‐scale studies are needed to uncover such associations with the date/delay effect.

Overall, one may speculate that in general, people are influenced by their time perception but only people with a lower date/delay effect are able to compensate for the effect at least to some extent, for example by exerting cognitive control and converting dates into delay units (at least approximately). On the other hand, people with a higher date/delay effect are influenced more strongly by differential time estimation itself, leading to lower discount rates in the date compared to the delay condition. It remains open how individuals with a higher date/delay effect adapt their strategy to the new requirements of the date condition, that is, whether, for instance, they ultimately rely more on rewards or use a different heuristic to avoid complex transformations.

### 4.2 Limitations

Our study has several limitations. First, our eye‐tracking sample (after exclusions) was rather small, the accuracy estimate of eye‐tracking data was not high, and effects concerning time attributes were potentially confounded by different reading requirements in the two conditions (i.e., dates are longer than delay units). While the replication of several effects of our previous study with a similar experimental task speaks for the validity of the data (Keidel, Murawski, & Ettinger, 2023), eye‐tracking results should only be seen as tentative evidence. Future studies should therefore aim to replicate and extend our results in larger samples. Additionally, given that we excluded several participants because they did not look at the time attribute of the SIR in a sufficient number of trials, future studies should consider varying the time attribute of the smaller‐sooner option as well (e.g., manipulate the number of days between 0 and a couple of days in the future). Importantly, while it was not possible to statistically control for reading confounds in our study due to the perfect correlation of condition with the number of symbols within time AOIs, future eye‐tracking studies could use different presentations of time attributes. For instance, using a different date format (e.g., “23/12/21”) may increase comparability with the delay condition (“29 days”) in terms of the number of symbols displayed. Additionally, studies could use a different delay format (e.g., 0 m/4w/1d, i.e., 0 months, 4 weeks, 1 day, vs. 23/12/21; or, supposing that all dates are in the same year, “29 d” vs. “23/12”) to parallelise the number of characters used in the two conditions. While we aimed to use a typical presentation form of dates in our study, such experimental manipulations could substantiate our findings while controlling for reading confounds.

Second, the use of the MCQ made it impossible to calculate SVs in the same way as in previous studies (Kable & Glimcher, 2007; Peters & Büchel, 2010), where a fixed reward was set and the SV was computed only for the presented delayed reward. Our intention was to examine the date/delay effect in a design comparable to previous studies, that is, with varying monetary amounts and delays (e.g., Dshemuchadse et al., 2013; Klapproth, 2012; Naudé et al., 2018), to ensure successful replication and higher generalisability. However, this may have weakened potential SV signals in our data. As previous findings suggest that the date/delay effect is robust even with simpler designs (LeBoeuf, 2006; Read et al., 2005), future studies could use a different task to gain higher comparability with other studies regarding SV signals and to be able to independently dissociate parametric effects of reward and time in the date/delay effect. We note, however, that in case of comparative strategies as implied by attribute‐based models (Amasino et al., 2019; Dai & Busemeyer, 2014; Marzilli Ericson et al., 2015; Zhang et al., 2022), this approach might not be valid for the date condition.

Third, our fMRI analysis approach was largely explorative, though preregistered. Thus, replication of our BOLD findings is needed.

## 5 CONCLUSION

Our study is the first to examine the date/delay effect using fMRI. Combining this approach with eye‐tracking, we found evidence for the differential time estimation hypothesis and the choice strategy hypothesis (Read et al., 2005; Zauberman et al., 2009). First, the date condition did not only lead to longer fixation durations on time versus reward attributes compared to the delay condition but we also found higher activity in precuneus/PCC and angular gyrus, that is, areas associated with episodic thinking, prospection and lower‐level construals (Lee et al., 2022; Schacter et al., 2007; Stillman et al., 2017; Tamir & Mitchell, 2011). Second, we found more comparative eye movements between time attributes in the date compared to the delay condition and higher prefrontal activity in the date > delay contrast in people with a lower date/delay effect, suggesting that people with a higher date/delay effect exert less cognitive control and calculate relatively less than others in the date condition (Arsalidou & Taylor, 2011; Niendam et al., 2012). In sum, our study suggests that the date/delay effect is contributed to by differential time estimation and some heuristic facilitating intertemporal choices. Future studies should examine more closely whether this heuristic is reflected, for instance, in higher weighting of reward differences or in some other cognitive process.

## CONFLICT OF INTEREST STATEMENT

The authors declare that they have no conflict of interest.

## Supporting information


**Data S1.** Supporting information

## Data Availability

The behavioural data that support the findings of this study are openly available in OSF at https://osf.io/td5gf/. The MRI data are available on request from the corresponding author. The MRI data are not publicly available due to ethical restrictions.

## Footnotes

^1^
We note that we deviated from the preregistration here, as we initially planned to use Bayes factors to compare model fits. However, recent evidence suggests that LOO‐CV yields the best‐approximating model and the most accurate parameter estimations (Lartillot, 2023).

^2^
Note that, using the delay > date contrast, the interpretation would be vice versa, that is, a higher date/delay effect was associated with higher activity differences in these regions.
