# Supplementary material for: The date/delay effect in intertemporal choice: A combined fMRI and eye‐tracking study
Source: Hum Brain Mapp. 2024 Feb 24;45(3):e26585. doi: 10.1002/hbm.26585 (PMC10893971; doi:10.1002/hbm.26585)
Supplement: Supplementary file 1 — Data S1. Supporting information [file HBM-45-e26585-s001.docx]

**The Date/Delay Effect in Intertemporal Choice: A Combined fMRI and Eye-Tracking Study**

**– Supplementary Material**

Kristof Keidel^1,2^[
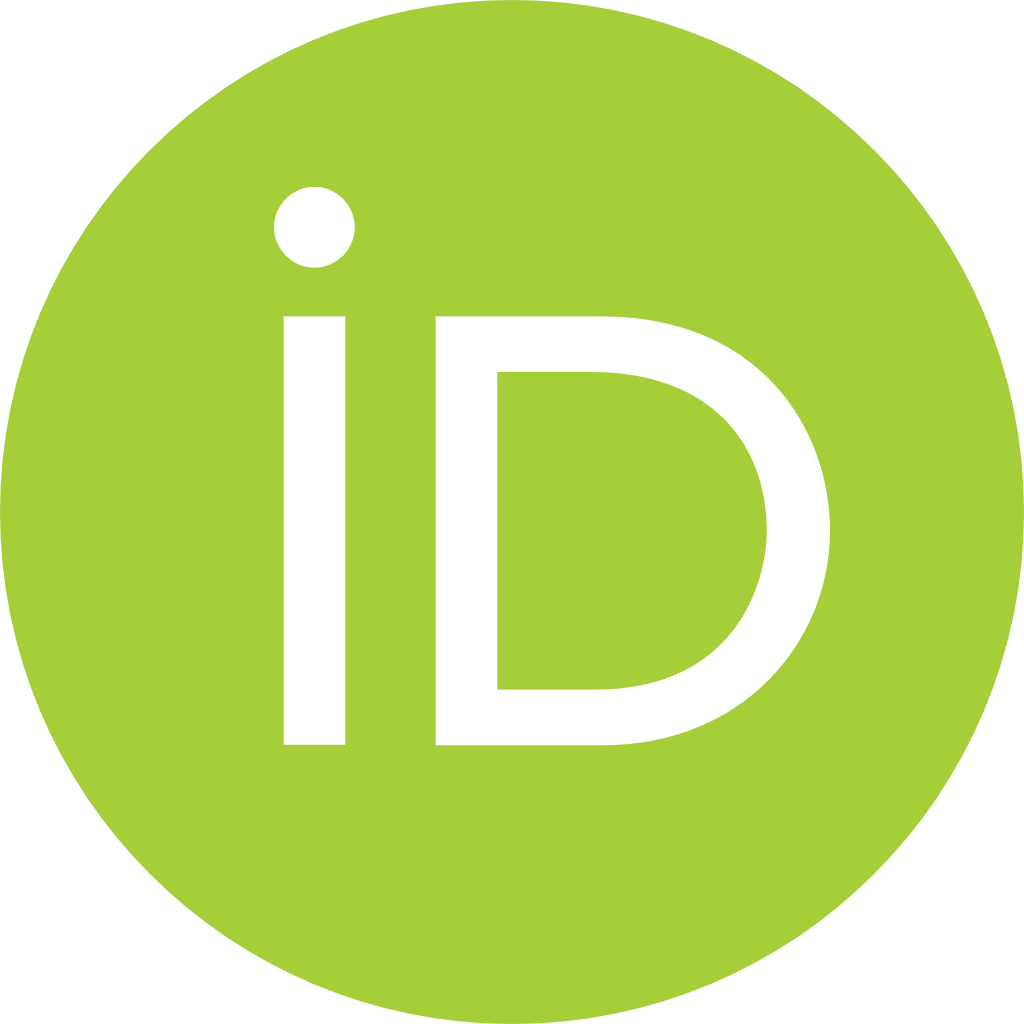
](https://orcid.org/0000-0002-1561-0157), Rebekka Schröder^1^[
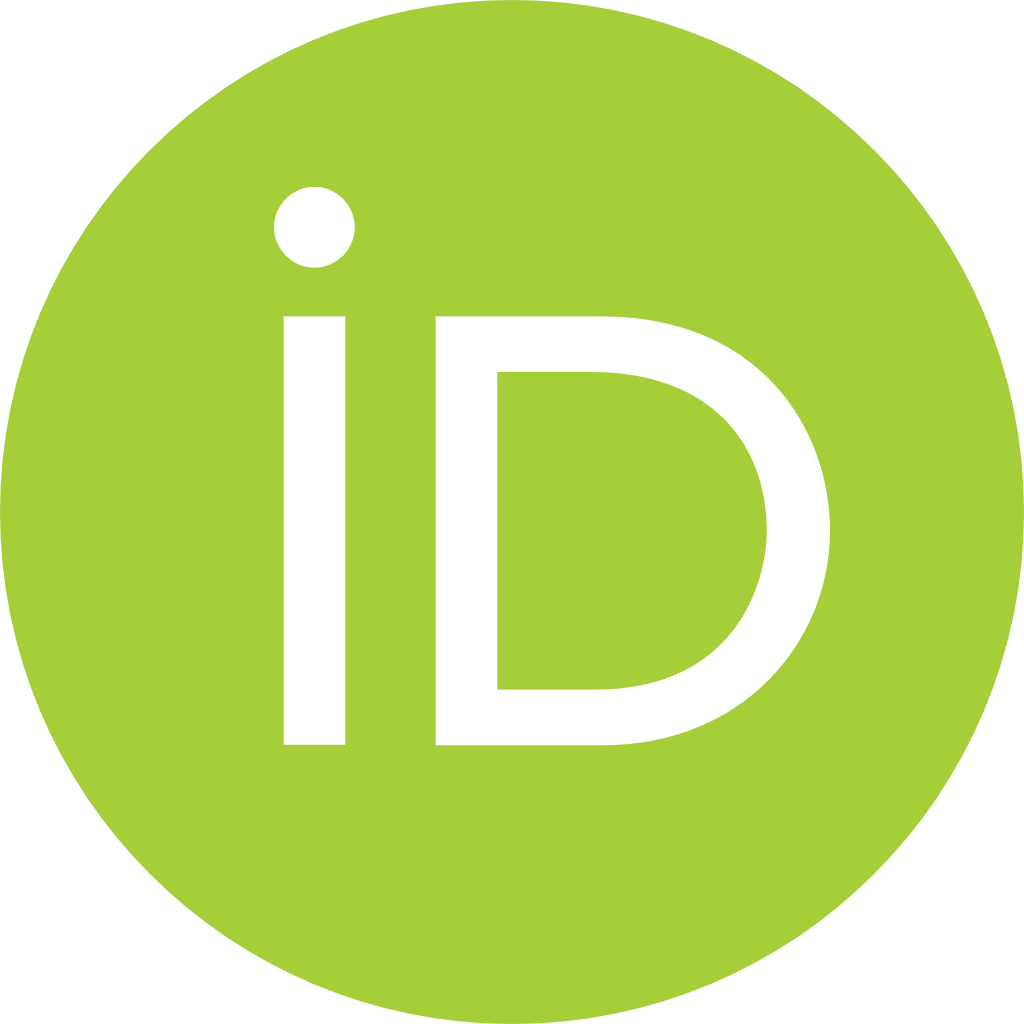
](https://orcid.org/0000-0002-0206-4484), Peter Trautner^3^, Alexander Radbruch^4,5^[
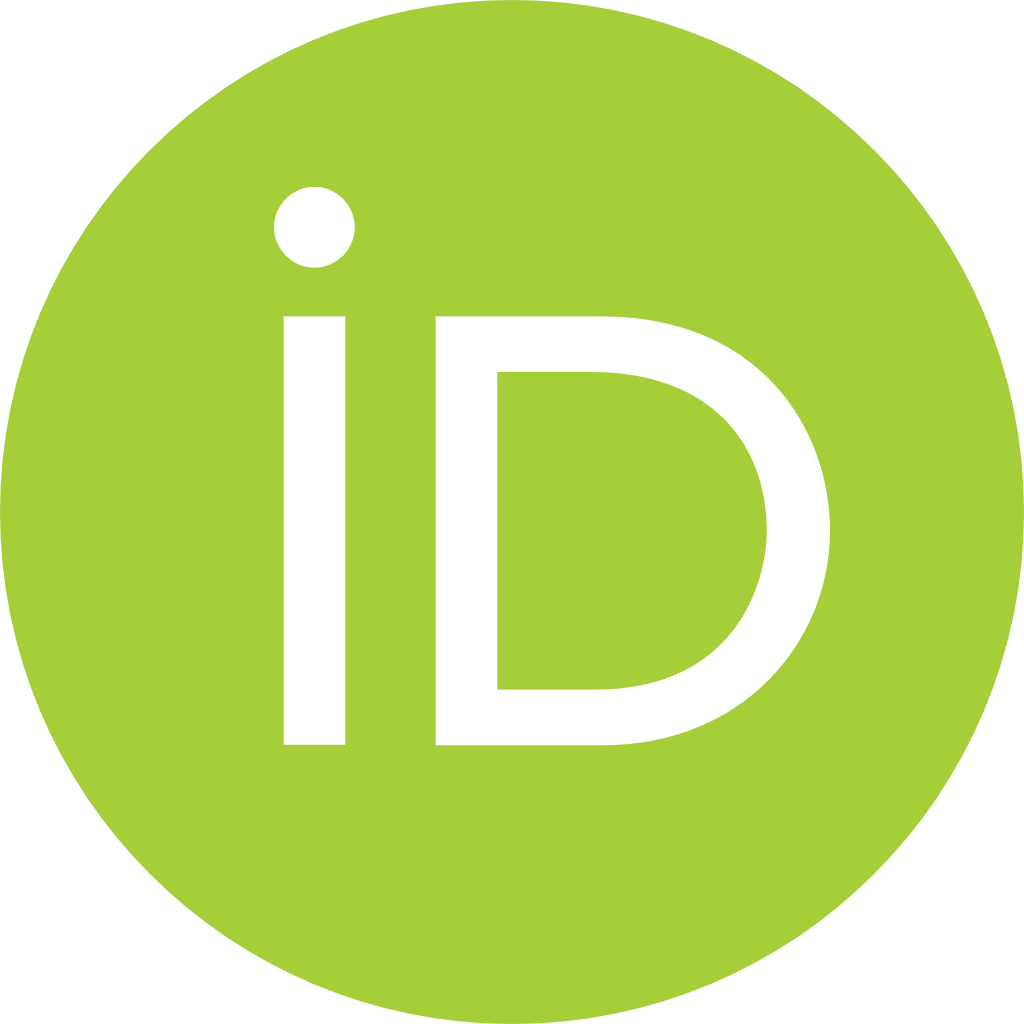
](https://orcid.org/0000-0001-6238-6525), Carsten Murawski^2^[
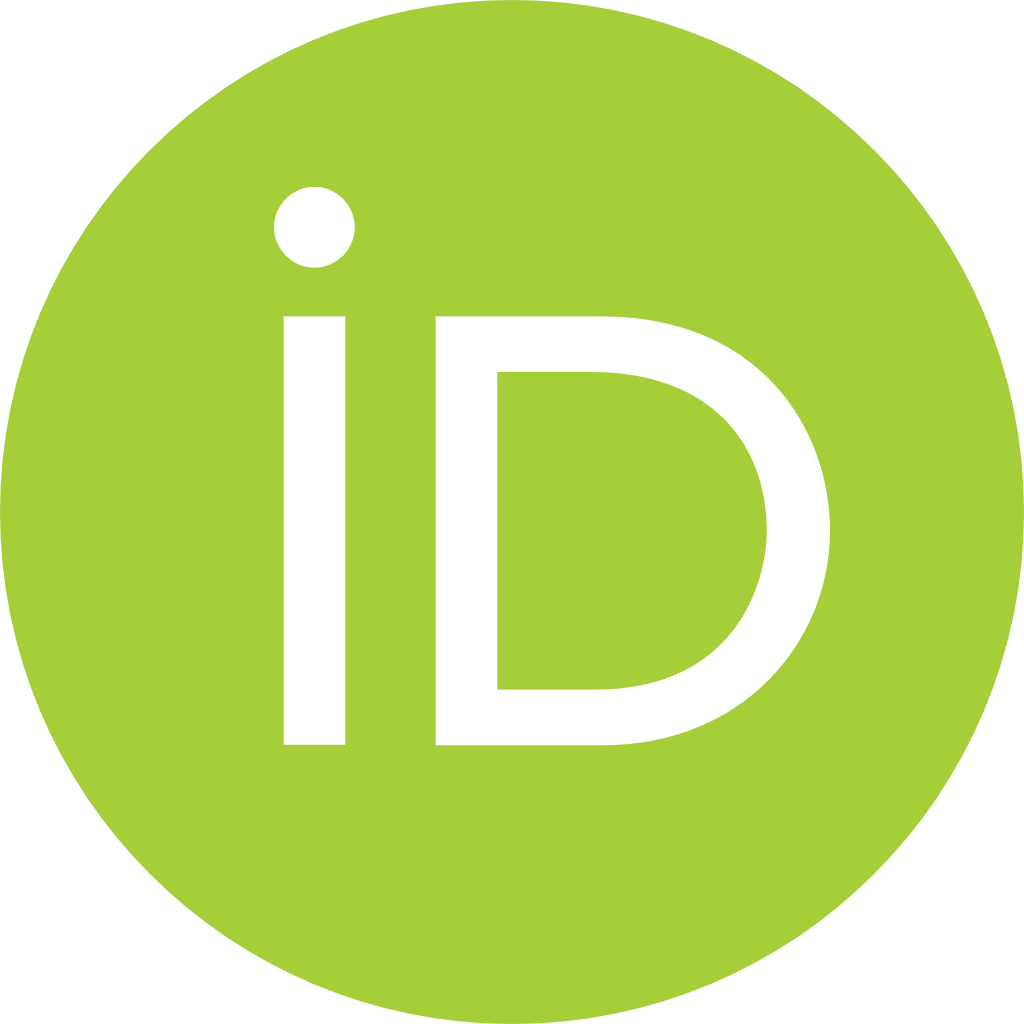
](https://orcid.org/0000-0002-1237-9535), and Ulrich Ettinger^1*^[
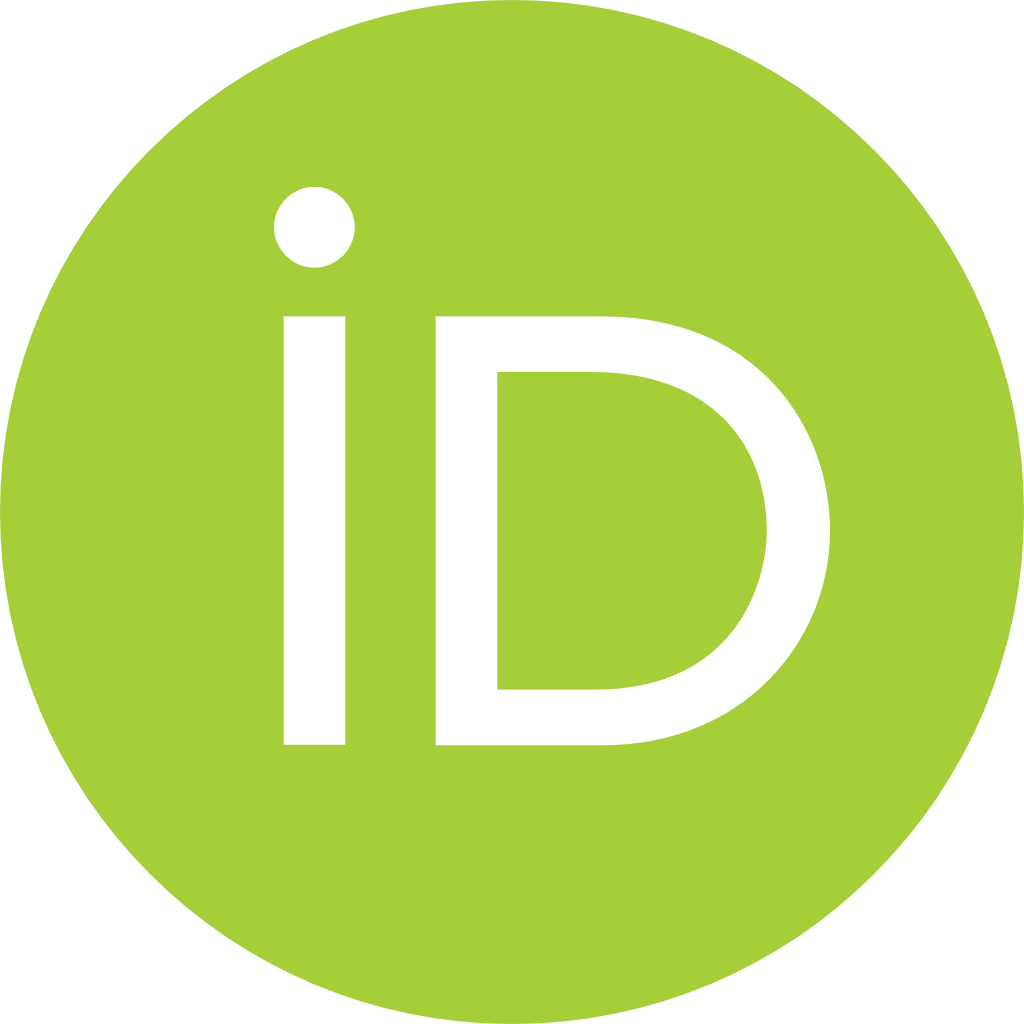
](https://orcid.org/0000-0002-0160-0281)

^1^ Department of Psychology, University of Bonn, Bonn, Germany

^2^ Centre for Brain, Mind and Markets, Department of Finance, The University of Melbourne, Carlton, Victoria, Australia

^3^ Core Facility Human 3T MRI, University of Bonn, Bonn, Germany

^4^ Clinic of Neuroradiology, University Hospital, Bonn, Germany

^5^ Clinical Neuroimaging, German Center for Neurodegenerative Diseases (DZNE), Bonn, Germany

# Supplement A: Discounting Rates ln(*k*) in the two Conditions per Participant


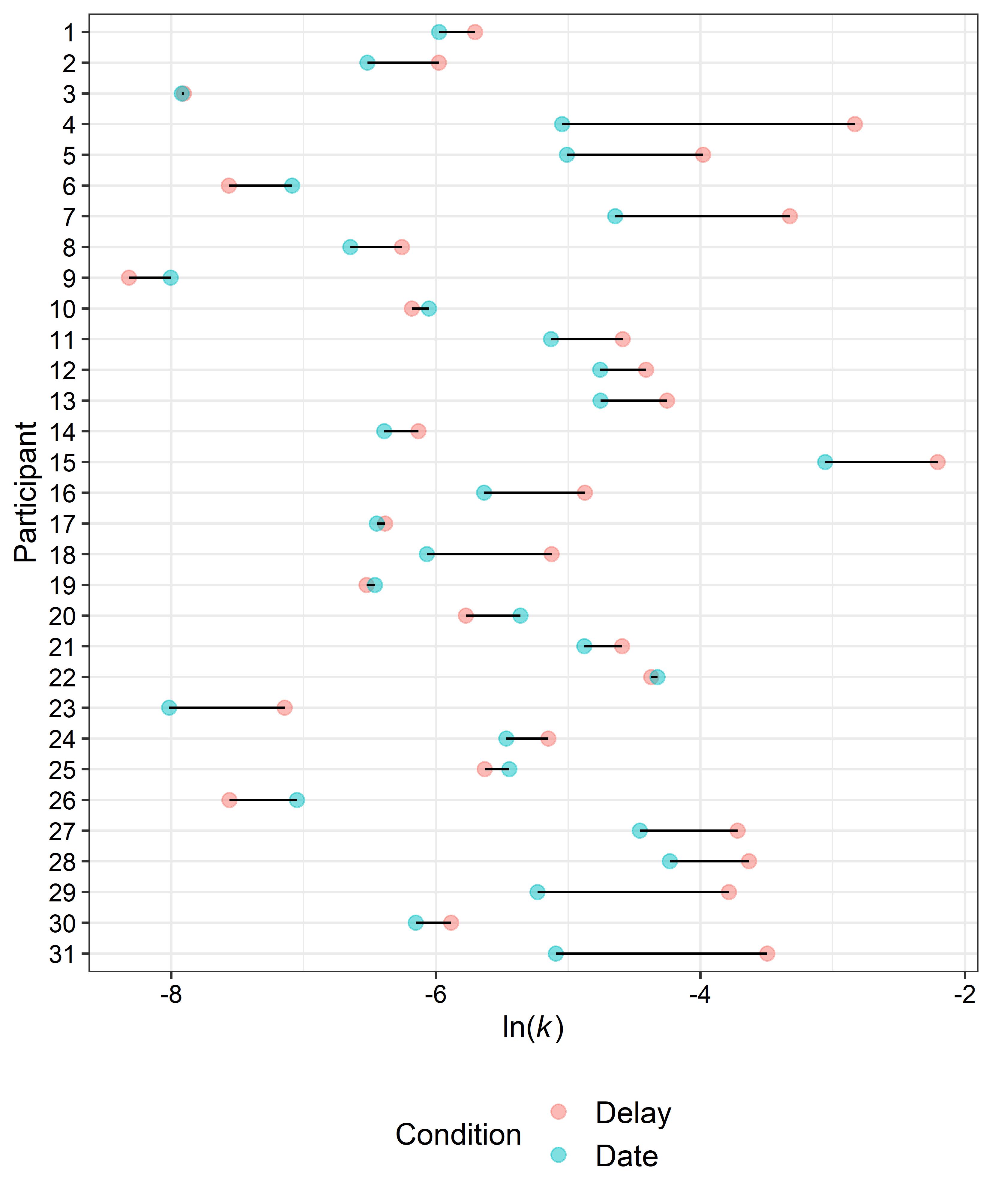


*Note*. The participant number corresponds to the order of recruitment.

# Supplement B: Analysis of Eye Movement Metrics Depending on Condition, Attribute Type and Option

## Table B1

*ANOVA for the Dependent Variable Duration of Fixations*

| Effect | df | *F* | *p* | η_p_² |
| --- | --- | --- | --- | --- |
| Condition | 1, 15 | 17.38 | <.001 | 0.54 |
| Attribute type | 1, 15 | 24.04 | <.001 | 0.62 |
| Option | 1, 15 | 38.27 | <.001 | 0.72 |
| Condition×Attribute type | 1, 15 | 103.74 | <.001 | 0.87 |
| Condition×Option | 1, 15 | 2.08 | .170 | 0.12 |
| Attribute type×Option | 1, 15 | 15.92 | .001 | 0.52 |
| Condition×Attribute type×Option | 1, 15 | 3.43 | .084 | 0.19 |

## Figure B2

*Number of Saccades (A) and Fixations (B) Depending on Condition (Date vs. Delay), Attribute Type (Reward vs. Time) and Option (Smaller-Immediate Reward, Larger-Later Reward)*


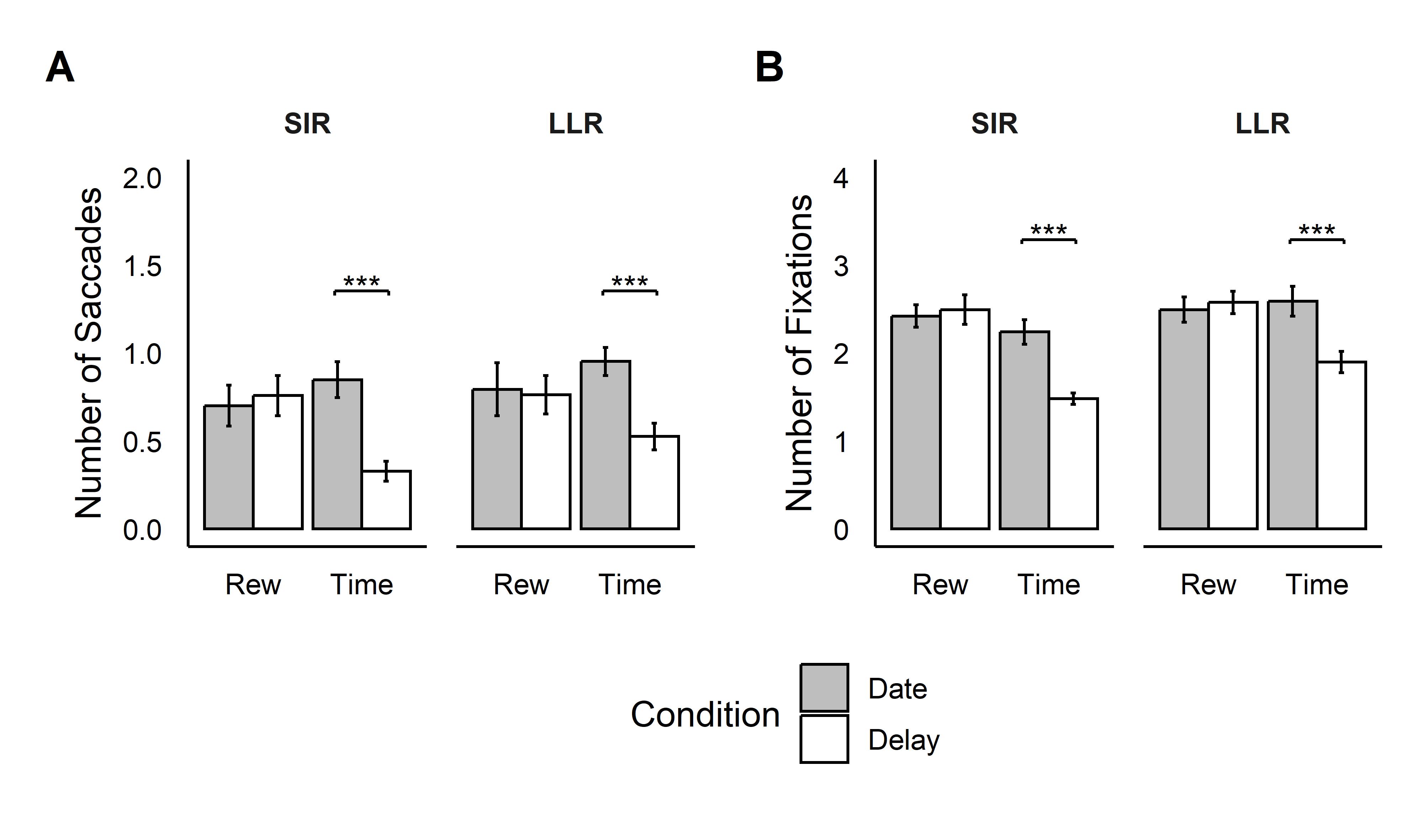


*Note*. Asterisks are only depicted for post-hoc t-tests within the same category (i.e., bars next to each other). Rew=Reward, SIR=Smaller-Immediate Reward, LLR=Larger-Later Reward.

*** *p*_adj_<.001 (Bonferroni-Holm corrected for multiple comparisons)

## Table B3

*ANOVAs for the Dependent Variables Number of Saccades and Number of Fixations*

| Effect | Number of Saccades (*df*=1,16) | | |  | | Number of Fixations (*df*=1,17) | | |  |
| --- | --- | --- | --- | --- | --- | --- | --- | --- | --- |
|  | *F* | *p* | η_p_² |  |  | *F* | *p* | η_p_² |  |
| Condition | 67.20 | <.001 | 0.81 |  |  | 32.27 | <.001 | 0.66 |  |
| Attribute type | 0.44 | .516 | 0.03 |  |  | 21.59 | <.001 | 0.56 |  |
| Option | 11.01 | .004 | 0.41 |  |  | 22.37 | <.001 | 0.57 |  |
| Condition×Attribute type | 28.69 | <.001 | 0.64 |  |  | 41.47 | <.001 | 0.71 |  |
| Condition×Option | 0.00 | .973 | 0.00 |  |  | 0.18 | .679 | 0.01 |  |
| Attribute type×Option | 1.05 | .320 | 0.06 |  |  | 11.50 | .003 | 0.40 |  |
| Condition×Attribute type×Option | 3.73 | .070 | 0.19 |  |  | 0.28 | .603 | 0.02 |  |

*Note*. Bonferroni-Holm corrected post-hoc *t*-tests exploring the crucial condition×attribute type interaction revealed that the difference between delay and date conditions in saccade number (time of smaller-immediate reward: *t*(16)=7.65, *p*_adj_<.001, *g*_av_=1.56; time of larger-later reward: *t*(16)=7.32, *p*_adj_<.001, *g*_av_=1.30) and fixation number (time of smaller-immediate reward: *t*(17)=7.38, *p*_adj_<.001, *g*_av_=1.72; time of larger-later reward: *t*(17)=6.19, *p*_adj_<.001, *g*_av_=1.09) is only significant for time attributes but not for reward attributes (all other *p*_adj_>.05).

# Supplement C: Descriptive Statistics of and Correlations Between Temporal Discounting and Eye Movement Variables

## Table C1

*Descriptive Statistics of and Correlations Between Temporal Discounting and Eye Movement Variables (Summary Measures)*

| Variable | *n* | *M* | *SD* | (1) | (2) | (3) | (4) | (5) | (6) | (7) | (8) | (9) | (10) | (11) | (12) | (13) | (14) |
| --- | --- | --- | --- | --- | --- | --- | --- | --- | --- | --- | --- | --- | --- | --- | --- | --- | --- |
| (1) ln(*k*) Delay | 18 | −5.24 | 1.60 | – |  |  |  |  |  |  |  |  |  |  |  |  |  |
| (2) ln(*k*) Date | 18 | −5.64 | 1.31 | .94*** | – |  |  |  |  |  |  |  |  |  |  |  |  |
| (3) D(ln(*k*)) | 17 | −0.29 | 0.40 | −.58* | −.36 | – |  |  |  |  |  |  |  |  |  |  |  |
| (4) *M*_RT_ Delay (s) | 18 | 2.37 | 0.52 | −.02 | .12 | −.12 | – |  |  |  |  |  |  |  |  |  |  |
| (5) *M*_RT_ Date (s) | 18 | 2.70 | 0.60 | .13 | .24 | −.30 | .90*** | – |  |  |  |  |  |  |  |  |  |
| (6) D(*M*_RT_) | 18 | 0.33 | 0.27 | .34 | .31 | −.39 | .06 | .49* | – |  |  |  |  |  |  |  |  |
| (7) CV_RT_ Delay | 18 | 0.25 | 0.07 | −.32 | −.29 | −.01 | .42 | .37 | .02 | – |  |  |  |  |  |  |  |
| (8) CV_RT_ Date | 18 | 0.24 | 0.07 | −.68** | −.68** | .03 | .20 | .12 | −.14 | .71*** | – |  |  |  |  |  |  |
| (9) D(CV) | 17 | −0.02 | 0.04 | −.45 | −.46 | .13 | −.21 | −.31 | −.28 | −.45 | .10 | – |  |  |  |  |  |
| (10) % AOI ch_ver_ Delay | 18 | 0.17 | 0.26 | .68** | .67** | −.39 | .07 | .27 | .47* | −.38 | −.65** | −.29 | – |  |  |  |  |
| (11) % AOI ch_ver_ Date | 18 | 0.07 | 0.22 | .55* | .61** | −.47 | .25 | .42 | .45 | −.25 | −.49* | −.13 | .85*** | – |  |  |  |
| (12) D(AOI ch_ver_) | 18 | −0.10 | 0.14 | −.42 | −.31 | −.01 | .28 | .16 | −.18 | .32 | .45 | .34 | −.55* | −.02 | – |  |  |
| (13) % Fix_rewdur_ Delay | 18 | 0.23 | 0.08 | −.35 | −.39 | −.36 | .18 | .06 | −.22 | .37 | .55* | .13 | −.49* | −.26 | .52* | – |  |
| (14) % Fix_rewdur_ Date | 18 | 0.00 | 0.11 | −.28 | −.20 | −.09 | .39 | .24 | −.21 | .01 | .27 | .42 | −.38 | −.17 | .44 | .54* | – |
| (15) D(% Fix_rewdur_) | 18 | −0.23 | 0.09 | −.01 | .12 | .21 | .29 | .23 | −.05 | −.31 | −.18 | .37 | .00 | .03 | .05 | −.26 | .67** |

*Note*. The table represents only data from participants whose eye-tracking data was analysed. All D(x) represent difference measures and are computed as difference of the variables given in the two lines above (date minus delay). *n* varies because of excluded outliers between 17 and 18. ln(*k*)=discount rate; RT=reaction time; CV=coefficient of variation (mean/standard deviation); % AOI ch_ver_=proportion of vertical saccades (relative to horizontal saccades) between areas of interest (Payne index); % Fix_rewdur_=proportion of fixation duration on reward attributes (relative to time attributes)

* *p*<.05, ** *p*<.01, *** *p*<.001

## Table C2

*Descriptive Statistics of and Correlations Between Temporal Discounting and Eye Movement Variables (More Specific Measures)*

| Variable | *n* | *M* | *SD* | (1) | (2) | (3) | (4) | (5) | (6) | (7) | (8) | (9) | (10) | (11) | (12) | (13) | (14) |
| --- | --- | --- | --- | --- | --- | --- | --- | --- | --- | --- | --- | --- | --- | --- | --- | --- | --- |
| (1) ln(*k*) Delay | 18 | −5.24 | 1.60 | – |  |  |  |  |  |  |  |  |  |  |  |  |  |
| (2) ln(*k*) Date | 18 | −5.64 | 1.31 | .94*** | – |  |  |  |  |  |  |  |  |  |  |  |  |
| (3) D(ln(*k*)) | 17 | −0.29 | 0.40 | −.58* | −.36 | – |  |  |  |  |  |  |  |  |  |  |  |
| (4) Hor-Rew Delay | 18 | 1.22 | 0.50 | −.53* | −.49* | .26 | – |  |  |  |  |  |  |  |  |  |  |
| (5) Hor-Rew Date | 18 | 1.29 | 0.38 | −.52* | −.53* | .08 | .81*** | – |  |  |  |  |  |  |  |  |  |
| (6) Hor-Time Delay | 18 | 0.66 | 0.27 | −.60** | −.49* | .55* | .70** | .68** | – |  |  |  |  |  |  |  |  |
| (7) Hor-Time Date | 18 | 1.00 | 0.29 | −.63** | −.58* | .58* | .68** | .64** | .77*** | – |  |  |  |  |  |  |  |
| (8) Ver-SIR Delay | 18 | 1.15 | 0.31 | .45 | .52* | −.23 | −.02 | .02 | −.29 | −.25 | – |  |  |  |  |  |  |
| (9) Ver-LL Delay | 18 | 1.40 | 0.40 | .40 | .54* | −.09 | −.09 | −.20 | −.34 | −.31 | .71** | – |  |  |  |  |  |
| (10) Ver-SIR Date | 18 | 1.27 | 0.45 | .53* | .62** | −.46 | −.21 | −.13 | −.52* | −.45 | .76*** | .78*** | – |  |  |  |  |
| (11) Ver-LL Date | 18 | 1.40 | 0.46 | .06 | .13 | −.26 | .10 | .24 | −.24 | −.10 | .63** | .67** | .75*** | – |  |  |  |
| (12) Dur-Rew Delay (ms) | 18 | 1094.69 | 268.11 | −.21 | −.10 | −.13 | .50* | .40 | .23 | .13 | .51* | .41 | .39 | .44 | – |  |  |
| (13) Dur-Rew Date (ms) | 17 | 995.59 | 233.52 | −.38 | −.26 | −.19 | .34 | .45 | .25 | .07 | .36 | .29 | .44 | .51* | .84*** | – |  |
| (14) Dur-Time Delay (ms) | 18 | 684.17 | 165.12 | −.06 | .12 | .15 | .21 | .22 | .13 | .09 | .68** | .68** | .51* | .63** | .76*** | .63** | – |
| (15) Dur-Time Date (ms) | 18 | 1036.25 | 256.48 | .14 | .23 | −.27 | .03 | .10 | −.20 | .03 | .68** | .65** | .68** | .70** | .65** | .62** | .75*** |

*Note*. The table represents only data from participants whose eye-tracking data was analysed. All D(x) represent difference measures and are computed as difference of the variables given in the two lines above (date minus delay). *n* varies because of excluded outliers between 17 and 18. ln(*k*)=discount rate; Hor-Rew=horizontal saccades between reward attributes; Hor-Time=horizontal saccades between time attributes; Ver-SIR=vertical saccades between smaller-immediate reward (SIR) attributes; Ver-LLR=vertical saccades between larger-later reward (LLR) attributes

* *p*<.05, ** *p*<.01, *** *p*<.001

# Supplement D: Descriptive Statistics of and Correlations Between Temporal Discounting and UPPS-P Variables

| Variable | *M* | *SD* | Skew | (1) | (2) | (3) | (4) | (5) | (6) | (7) | (8) |
| --- | --- | --- | --- | --- | --- | --- | --- | --- | --- | --- | --- |
| (1) ln(*k*) Delay | −5.27 | 1.55 | −0.10 | – |  |  |  |  |  |  |  |
| (2) ln(*k*) Date | −5.72 | 1.17 | −0.21 | .93*** | – |  |  |  |  |  |  |
| (3) D(ln(*k*)) | −0.45 | 0.64 | −0.67 | −.72*** | −.41* | – |  |  |  |  |  |
| (4) Positive Urgency | 22.32 | 5.97 | 0.93 | −.04 | −.01 | .07 | (.85) |  |  |  |  |
| (5) Negative Urgency | 23.77 | 5.70 | 0.04 | −.22 | −.15 | .26 | .64*** | (.78) |  |  |  |
| (6) Sensation Seeking | 39.48 | 7.83 | −0.34 | −.06 | −.06 | .02 | .34 | .33 | (.80) |  |  |
| (7) Lack of Premeditation | 24.65 | 6.15 | −0.15 | .10 | .11 | −.03 | .08 | .00 | .00 | (.82) |  |
| (8) Lack of Perseverance | 19.13 | 4.96 | 0.70 | −.09 | −.01 | .20 | .42* | .32 | .05 | .42* | (.82) |

*Note*. The date/delay effect D(ln(*k*)) is computed as ln(*k*) Date − ln(*k*) Delay. Values in the correlation diagonal represent Cronbach’s Alphas. Correlations: *df* = 29. *M* = mean; *SD* = standard deviation; Skew = Skewness; ln(*k*) = log-transformed discount rate

* *p*<.05, ** *p*<.01, *** *p*<.001 (unadjusted)

# Supplement E: Linear mixed-model Predicting Choice Behaviour by Condition and Differences in Reward and Time Values

| Variable | *Estimate* | *SE* | *p* |
| --- | --- | --- | --- |
| Constant | 0.45 | 0.46 | .331 |
| Condition | 0.44 | 0.12 | < .001 |
| Δreward | 1.86 | 0.21 | < .001 |
| Δtime | −2.94 | 0.20 | < .001 |
| Condition ×Δreward | 0.19 | 0.20 | .334 |
| Condition ×Δtime | −0.28 | 0.18 | .124 |
| Δreward×Δtime | −0.11 | 0.23 | .631 |
| Condition×Δreward×Δtime | −0.20 | 0.21 | .330 |

*Note*. Choices are coded 0 for smaller-immediate and 1 for larger-later rewards. Condition (effect coded: −1=delay, 1=date), difference in reward (Δreward) and difference in time (Δtime) are modelled as fixed effects. Random effects for participants are included for the constant.

# Supplement F: Additional BOLD Activity Tables (Main Contrasts)

## Table F1

*BOLD Response in Delay vs. Baseline*

| **Anatomical Label (***Functional Label)* | **Cluster size** | ***t*-value** | **MNI coordinates** | | |
| --- | --- | --- | --- | --- | --- |
|  |  |  | **x** | **y** | **z** |
| L Calcarine Gyrus (*V1*) | 22,567 | 11.20 | −10 | −72 | 12 |
| R Superior Occipital Gyrus |  | 11.05 | 26 | −68 | 28 |
| R ACC |  | 10.67 | 12 | 22 | 32 |
| R ACC |  | 10.60 | 10 | 20 | 34 |
| L Fusiform Gyrus (*FG4*) |  | 10.60 | −40 | −54 | −12 |
| R Inferior Occipital Gyrus (*hOc4la*) |  | 10.37 | 42 | −80 | −6 |
| N/A^a^ |  | 10.20 | −30 | −48 | 46 |
| R MCC |  | 10.10 | 6 | 14 | 44 |
| R Cerebellum (*V4*) |  | 9.73 | 34 | −84 | −14 |
| L MCC |  | 9.69 | −4 | 6 | 48 |
| R Lingual Gyrus |  | 9.49 | 12 | −78 | 8 |
| R IFG | 726 | 9.62 | 34 | 26 | −6 |
| R IFG |  | 9.44 | 38 | 18 | −4 |
| L Insula Lobe | 606 | 9.86 | −28 | 22 | 0 |
| L Insula Lobe |  | 8.35 | −36 | 12 | 2 |
| L IFG | 529 | 8.18 | −42 | 2 | 32 |
| L Precentral Gyrus (*Area 44*) |  | 7.75 | −56 | 4 | 36 |
| L Precentral Gyrus (*Area 44*) |  | 7.58 | −58 | 6 | 34 |
| L IFG (*Area 44*) |  | 7.38 | −56 | 4 | 24 |
| R IFG | 248 | 7.78 | 44 | 8 | 30 |
| R IFG | 98 | 7.10 | 46 | 28 | 22 |
| R Caudate Nucleus | 58 | 7.24 | 14 | 10 | 0 |
| R Middle Frontal Gyrus | 10 | 6.04 | 30 | 34 | 30 |
| L Putamen | 10 | 6.08 | −16 | 0 | −2 |
| L Putamen |  | 5.87 | −16 | 4 | 0 |
| L Putamen |  | 5.78 | −16 | 8 | 0 |

*Note*. The Anatomy Toolbox (Eickhoff et al., 2005) was used to infer anatomical labels. In case of an unknown area (N/A), we additionally specified the region in the footnote using the Neuromorphometrics atlas in SPM. Cluster size is given in number of voxels. MNI = Montreal Neurological Institute

^a^ L Superior Parietal Lobule

## Table F2

*BOLD Response in Date vs. Baseline*

| **Anatomical Label (***Functional Label)* | **Cluster size** | ***t*-value** | **MNI coordinates** | | |
| --- | --- | --- | --- | --- | --- |
|  |  |  | **x** | **y** | **z** |
| R Superior Occipital Gyrus | 27,537 | 11.91 | 26 | −68 | 30 |
| L Lingual Gyrus (*V1*) |  | 11.86 | −10 | −70 | 10 |
| L Lingual Gyrus (*V1*) |  | 11.64 | −8 | −74 | 12 |
| R Calcarine Gyrus (*V1*) |  | 11.07 | 12 | −80 | 8 |
| L Lingual Gyrus (*V3v*) |  | 11.06 | −10 | −86 | −2 |
| R ACC |  | 10.87 | 10 | 22 | 32 |
| R MCC |  | 10.58 | 8 | 14 | 46 |
| R Calcarine Gyrus (*V1*) |  | 10.28 | 10 | −72 | 12 |
| L Cerebellum (*FG1*) |  | 10.26 | −30 | −74 | −14 |
| N/A^a^ |  | 10.14 | −30 | −46 | 44 |
| L Cerebellum (*V4*) |  | 10.04 | −16 | −78 | −8 |
| L Insula Lobe | 573 | 9.47 | −28 | 22 | 0 |
| L Insula Lobe |  | 7.16 | −38 | 12 | 4 |
| R IFG | 226 | 8.46 | 44 | 10 | 32 |
| R IFG |  | 6.27 | 40 | 2 | 36 |
| R Precentral Gyrus |  | 6.06 | 52 | 0 | 38 |
| R IFG | 121 | 7.40 | 44 | 24 | 26 |
| N/A^b^ | 94 | 6.74 | −4 | −16 | 30 |
| N/A^c^ |  | 6.44 | 8 | −28 | 28 |
| N/A^d^ |  | 6.41 | 6 | −16 | 30 |
| N/A^d^ |  | 6.31 | 4 | −18 | 28 |
| N/A^e^ |  | 5.93 | −6 | −28 | 28 |
| R Middle Frontal Gyrus | 26 | 6.63 | 30 | 36 | 32 |
| R Cerebellum (*Lobule VI*) | 22 | 6.21 | 40 | −48 | −32 |
| L IFG | 19 | 6.47 | −42 | 26 | 28 |

*Note*. The Anatomy Toolbox (Eickhoff et al., 2005) was used to infer anatomical labels. In case of an unknown area (N/A), we additionally specified the region in the footnote using the Neuromorphometrics atlas in SPM. Cluster size is given in number of voxels. MNI = Montreal Neurological Institute

^a^ L IPS, ^b^ L Middle Cingulate Gyrus, ^c^ R Anterior Cingulate Gyrus,
^d^ R Middle Cingulate Gyrus, ^e^ L Posterior Cingulate Gyrus

# Supplement G: ROI Analyses

## Table G1

*Parametric Modulation Effects of Subjective Value (SV) in the Regions of Interest (ROIs) Ventral Striate (vS) and Ventro-Medial Prefrontal Cortex (vmPFC)*

| Regressor | ROI | Comparison | *t* | *p* | *Effect* |
| --- | --- | --- | --- | --- | --- |
| Sum of SVs | vS | Delay vs. 0 | 2.60 | .014 | 0.47 |
|  | vS | Date vs. 0 | −0.42 | .674 | 0.08 |
|  | vS | Delay vs. Date | 2.18 | .037 | 0.57 |
|  | vmPFC | Delay vs. 0 | 0.79 | .438 | 0.14 |
|  | vmPFC | Date vs. 0 | −0.05 | .964 | 0.01 |
|  | vmPFC | Delay vs. Date | 0.61 | .544 | 0.14 |
| SV of chosen option | vS | Delay vs. 0 | 3.26 | .003 | 0.59 |
|  | vS | Date vs. 0 | −0.12 | .906 | 0.02 |
|  | vS | Delay vs. Date | 2.25 | .032 | 0.60 |
|  | vmPFC | Delay vs. 0 | 0.94 | .355 | 0.17 |
|  | vmPFC | Date vs. 0 | 0.46 | .651 | 0.08 |
|  | vmPFC | Delay vs. Date | 0.32 | .748 | 0.07 |
| Difference of SVs (LLR−SIR) | vS | Delay vs. 0 | 0.81 | .427 | 0.14 |
|  | vS | Date vs. 0 | 0.61 | .545 | 0.11 |
|  | vS | Delay vs. Date | 0.17 | .866 | 0.04 |
|  | vmPFC | Delay vs. 0 | 1.46 | .154 | 0.26 |
|  | vmPFC | Date vs. 0 | 0.36 | .724 | 0.06 |
|  | vmPFC | Delay vs. Date | 0.69 | .493 | 0.18 |
| Ratio of SVs (LLR/SIR) | vS | Delay vs. 0 | −1.06 | .771 | 0.05 |
|  | vS | Date vs. 0 | 0.49 | .628 | 0.09 |
|  | vS | Delay vs. Date | −0.16 | .870 | 0.04 |
|  | vmPFC | Delay vs. 0 | 0.91 | .368 | 0.16 |
|  | vmPFC | Date vs. 0 | 1.02 | .315 | 0.18 |
|  | vmPFC | Delay vs. Date | 0.00 | .998 | 0.00 |

*Note*. SVs of answer options (LLR=larger-later reward; SIR=smaller-immediate reward) were computed using the individual discount functions for the delay and date conditions (for details, see main text). To present all possible modulations of SV, we used the sum of SVs, the SV of the chosen option, the difference of SVs and the ratio of SVs as parametric modulators of the delay and date conditions. Betas of both conditions were compared to zero (one-sample *t*-tests) and to each other (paired *t*-tests). Effects are presented as Cohen’s (*M*/*SD*) for one-sample *t*-tests against zero and as Hedge’s g_av_ for paired *t*-tests (delay vs. date). All *df*=30.

## Table G2

*Parametric Modulation Effects of Objective Rewards and Objective Times in the Regions of Interest (ROIs) Ventral Striate (vS) and Ventro-Medial Prefrontal Cortex (vmPFC)*

| Regressor | ROI | Comparison | *t* | *p* | *Effect* |
| --- | --- | --- | --- | --- | --- |
| Objective Rewards (LLR+SIR) | vS | Delay vs. 0 | 2.57 | .015 | 0.46 |
|  | vS | Date vs. 0 | −0.64 | .529 | 0.11 |
|  | vS | Delay vs. Date | 2.16 | .039 | 0.57 |
|  | vmPFC | Delay vs. 0 | 0.96 | .344 | 0.17 |
|  | vmPFC | Date vs. 0 | −0.03 | .977 | 0.01 |
|  | vmPFC | Delay vs. Date | 0.72 | .478 | 0.16 |
| Objective Times (LLR+SIR) | vS | Delay vs. 0 | −0.78 | .444 | 0.14 |
|  | vS | Date vs. 0 | 0.68 | .502 | 0.12 |
|  | vS | Delay vs. Date | −0.95 | .351 | 0.26 |
|  | vmPFC | Delay vs. 0 | −1.12 | .270 | 0.20 |
|  | vmPFC | Date vs. 0 | 0.05 | .963 | 0.01 |
|  | vmPFC | Delay vs. Date | −0.93 | .362 | .0.23 |
| Objective Rewards (LLR−SIR) | vS | Delay vs. 0 | 2.97 | .006 | 0.53 |
|  | vS | Date vs. 0 | 0.70 | .487 | 0.13 |
|  | vS | Delay vs. Date | 1.27 | .215 | 0.31 |
|  | vmPFC | Delay vs. 0 | 0.59 | .563 | 0.11 |
|  | vmPFC | Date vs. 0 | 0.34 | .738 | 0.06 |
|  | vmPFC | Delay vs. Date | 0.08 | .933 | 0.02 |
| Objective Times (LLR−SIR) | vS | Delay vs. 0 | 2.27 | .030 | 0.41 |
|  | vS | Date vs. 0 | 0.80 | .431 | 0.14 |
|  | vS | Delay vs. Date | 0.76 | .451 | 0.21 |
|  | vmPFC | Delay vs. 0 | −0.21 | .832 | 0.04 |
|  | vmPFC | Date vs. 0 | 0.21 | .837 | 0.04 |
|  | vmPFC | Delay vs. Date | −0.36 | .720 | 0.07 |

*Note*. Objective rewards and times of answer options (LLR=larger-later reward; SIR=smaller-immediate reward) for each trial were either subtracted from or added to each other and served as parametric modulators in separate models. For each variant, we used the automated orthogonalization in SPM and set up two separate models in which either reward or timing were entered first and the other entered second to obtain variances specific to the variable entered second. Betas of both conditions were compared to zero (one-sample *t*-tests) and to each other (paired *t*-tests). Effects are presented as Cohen’s (*M*/*SD*) for one-sample *t*-tests against zero and as Hedge’s g_av_ for paired *t*-tests (delay vs. date). All *df*=30.

# Supplement H: Clusters Showing Significant Correlations Between the Behavioural Date/Delay Effect and BOLD Signal Change in the Contrast Date > Delay

| **Anatomical Label (***Functional Label)* | **Cluster size** | ***t*-value** | **MNI coordinates** | | |
| --- | --- | --- | --- | --- | --- |
|  |  |  | **x** | **y** | **z** |
| N/A^a^ | 846 | 6.08 | 0 | −86 | −10 |
| R Cerebellum (*Lobule VI*) |  | 5.63 | 14 | −78 | −12 |
| Cerebellar Vermis |  | 4.47 | 0 | −66 | 4 |
| R Cerebellum (*Lobule V*) |  | 4.38 | 12 | −56 | −10 |
| Cerebellar Vermis (*L Lobule VI*) |  | 4.33 | 0 | −76 | −8 |
| Cerebellar Vermis |  | 4.29 | 6 | −56 | −12 |
| L Cerebellum (*Lobule V*) |  | 4.13 | −4 | −64 | −4 |
| Cerebellar Vermis (*L* *Lobule I IV*) |  | 4.13 | 0 | −56 | −10 |
| L Calcarine Gyrus (*V1*) |  | 4.11 | 2 | −94 | 10 |
| R Lingual Gyrus (*V2*) |  | 3.85 | 10 | −92 | −6 |
| R Lingual Gyrus (*V1*) |  | 3.72 | 6 | −60 | 14 |
| R Superior Frontal Gyrus | 834 | 5.65 | 30 | 60 | 16 |
| R IFG |  | 5.57 | 54 | 20 | 36 |
| R Middle Frontal Gyrus |  | 5.47 | 40 | 48 | 24 |
| R Middle Frontal Gyrus |  | 5.44 | 42 | 50 | 22 |
| R Middle Frontal Gyrus |  | 5.16 | 36 | 46 | 34 |
| R Middle Frontal Gyrus |  | 5.01 | 34 | 48 | 36 |
| R Middle Frontal Gyrus |  | 4.96 | 32 | 52 | 30 |
| R Superior Medial Gyrus (*Area Fp1*) |  | 4.67 | 10 | 64 | 12 |
| R Middle Frontal Gyrus |  | 4.63 | 50 | 28 | 36 |
| R IFG |  | 4.46 | 52 | 30 | 30 |
| R Superior Medial Gyrus |  | 4.34 | 14 | 56 | 38 |
| L MCC | 268 | 4.71 | −12 | 16 | 40 |
| L ACC |  | 4.63 | −2 | 32 | 20 |
| L ACC |  | 4.55 | 0 | 26 | 20 |
| L ACC |  | 4.46 | −2 | 20 | 32 |
| L ACC |  | 4.46 | −6 | 40 | 18 |
| L Superior Medial Gyrus |  | 4.44 | −6 | 30 | 44 |
| L Superior Medial Gyrus |  | 4.21 | −2 | 28 | 46 |
| L ACC |  | 4.17 | −6 | 36 | 22 |
| L Superior Medial Gyrus |  | 4.12 | −10 | 20 | 44 |
| L MCC |  | 3.95 | −8 | 20 | 38 |
| R MCC |  | 3.58 | 6 | 30 | 42 |
| L Middle Frontal Gyrus | 201 | 5.83 | −32 | 40 | 38 |
| L Middle Frontal Gyrus |  | 4.88 | −36 | 36 | 42 |
| L Middle Frontal Gyrus |  | 4.22 | −30 | 50 | 32 |
| R Posterior-Medial Frontal | 176 | 4.74 | 10 | 18 | 60 |
| R Posterior-Medial Frontal |  | 4.19 | 10 | 14 | 64 |
| L Posterior-Medial Frontal |  | 4.12 | −2 | 12 | 64 |
| R Posterior-Medial Frontal |  | 3.98 | 6 | 14 | 62 |
| R Superior Medial Gyrus |  | 3.97 | 6 | 26 | 56 |

*Note*. The Anatomy Toolbox (Eickhoff et al., 2005) was used to infer anatomical labels. In case of an unknown area (N/A), we additionally specified the region in the footnote using the Neuromorphometrics atlas in SPM. Cluster size is given in number of voxels. MNI = Montreal Neurological Institute

^a^ L Lingual Gyrus

# Supplement I: Descriptive Statistics of and Correlations Between Temporal Discounting and UPPS-P Variables

## Table I1

*Pearson Correlations Between the Date/Delay Effect, Significant BOLD Clusters in the Contrast Date > Delay and Eye-Tracking Measures*

| Variable | *n* | *M* | *SD* | Skew | (1) | (2) | (3) | (4) | (5) | (6) | (7) | (8) |
| --- | --- | --- | --- | --- | --- | --- | --- | --- | --- | --- | --- | --- |
| (1) D(ln(*k*)) | 17 | −0.29 | 0.40 | 0.30 | – |  |  |  |  |  |  |  |
| (2) D(AOI ch_ver_) | 18 | −0.10 | 0.14 | −0.48 | −.01 | – |  |  |  |  |  |  |
| (3) D(% Fix_rewdur_) | 18 | −0.23 | 0.09 | −0.20 | .21 | .05 | – |  |  |  |  |  |
| (4) L/R Occipital Areas | 18 | −0.41 | 0.27 | −0.04 | −.29 | .18 | −.50* | – |  |  |  |  |
| (5) R Precuneus | 18 | −0.34 | 0.32 | 0.20 | −.15 | −.11 | −.10 | .33 | – |  |  |  |
| (6) L Precuneus | 18 | −0.44 | 0.35 | 0.09 | −.02 | .21 | −.16 | .28 | .84*** | – |  |  |
| (7) R Angular Gyrus | 17 | −0.48 | 0.42 | 0.00 | .02 | .03 | −.14 | .20 | .64** | .65** | – |  |
| (8) L Angular Gyrus | 18 | −0.47 | 0.50 | −0.78 | −.12 | .15 | −.30 | .40 | .60** | .72*** | .85*** | – |
| (9) L Middle/Superior Frontal Gyrus | 18 | −0.16 | 0.15 | −0.64 | .16 | −.02 | −.14 | .37 | .46 | .49* | .54* | .58* |

*Note*. The table represents only data from participants whose eye-tracking data was analysed. All D(x) represent difference measures and are computed as difference of the variables given in the two lines above (date minus delay). *n* varies because of excluded outliers between 17 and 18. % AOI ch_ver_=proportion of vertical saccades (relative to horizontal saccades) between areas of interest; % Fix_rewdur_=proportion of fixation duration on reward attributes (relative to time attributes). Skew = Skewness

* *p*<.05, ** *p*<.01, *** *p*<.001 (unadjusted)

## Table I2

*Pearson Correlations Between the Date/Delay Effect, Significant BOLD Clusters in the Contrast Date > Delay and Eye-Tracking Measures*

| Variable | *n* | *M* | *SD* | Skew | (1) | (2) | (3) | (4) | (5) | (6) | (7) |
| --- | --- | --- | --- | --- | --- | --- | --- | --- | --- | --- | --- |
| (1) D(ln(*k*)) | 17 | −0.29 | 0.40 | 0.30 | – |  |  |  |  |  |  |
| (2) D(AOI ch_hor_) | 18 | −0.10 | 0.14 | −0.48 | −.01 | – |  |  |  |  |  |
| (3) D(% Fix_rewdur_) | 18 | −0.23 | 0.09 | −0.20 | .21 | .05 | – |  |  |  |  |
| (4) L/R Occipital Areas | 17 | 0.11 | 0.38 | 0.03 | .74*** | −.01 | .04 | – |  |  |  |
| (5) L Middle Frontal Gyrus | 18 | 0.00 | 0.44 | −0.02 | .44 | .40 | .09 | .71** | – |  |  |
| (6) R Middle/Superior Frontal Gyrus | 17 | 0.16 | 0.39 | 0.62 | .39 | .12 | −.10 | .58* | .82*** | – |  |
| (7) L/R Posterior Medial Frontal Cortex | 18 | 0.06 | 0.35 | −0.50 | .53* | .21 | .38 | .59* | .76*** | .71** | – |
| (8) L/R Anterior/Mid Cingulate Cortex | 17 | −0.06 | 0.29 | 0.30 | .71** | −.14 | .13 | .80*** | .46 | .48* | .59* |

*Note*. The table represents only data from participants whose eye-tracking data was analysed. All D(x) represent difference measures and are computed as difference of date and delay conditions (date minus delay). *n* varies because of excluded outliers between 17 and 18. % AOI ch_ver_=proportion of vertical saccades (relative to horizontal saccades) between areas of interest; % Fix_rewdur_=proportion of fixation duration on reward attributes (relative to time attributes); Skew = Skewness

* *p*<.05, ** *p*<.01, *** *p*<.001 (unadjusted)

# References

Eickhoff, S. B., Stephan, K. E., Mohlberg, H., Grefkes, C., Fink, G. R., Amunts, K., & Zilles, K. (2005). A new SPM toolbox for combining probabilistic cytoarchitectonic maps and functional imaging data. *NeuroImage*, *25*(4), 1325–1335. https://doi.org/10.1016/j.neuroimage.2004.12.034
